# Investigating the association of NBN gene polymorphisms with multiple cancers through statistical meta-analysis and bioinformatics insights

**DOI:** 10.1093/biomethods/bpag012

**Published:** 2026-02-27

**Authors:** Md Harun-Or-Roshid, Md Selim Reza, Md Ariful Islam, Md Humaun Farid, Md Mostafizur Rahman, Saroje Kumar Sarkar, Md Nurul Haque Mollah

**Affiliations:** Laboratory of Bioinformatics, Department of Statistics, University of Rajshahi, Rajshahi 6205, Bangladesh; Laboratory of Bioinformatics, Department of Statistics, University of Rajshahi, Rajshahi 6205, Bangladesh; Division of Biomedical Informatics and Genomics, School of Medicine, Tulane University, New Orleans, LA 70112, United States; Laboratory of Bioinformatics, Department of Statistics, University of Rajshahi, Rajshahi 6205, Bangladesh; Laboratory of Bioinformatics, Department of Statistics, University of Rajshahi, Rajshahi 6205, Bangladesh; Laboratory of Bioinformatics, Department of Statistics, University of Rajshahi, Rajshahi 6205, Bangladesh; Laboratory of Bioinformatics, Department of Statistics, University of Rajshahi, Rajshahi 6205, Bangladesh; Laboratory of Bioinformatics, Department of Statistics, University of Rajshahi, Rajshahi 6205, Bangladesh

**Keywords:** NBN gene polymorphisms, rs1805794 and rs709816, multiple cancers, association studies, statistical meta-analysis, bioinformatics analysis

## Abstract

Several individual genetic association studies, including meta-analyses, have investigated the association of two SNPs (rs1805794 and rs709816) of NBN gene with multiple cancer risks. However, their findings were inconsistent, making it challenging to use NBN gene as a diagnostic and prognostic biomarker. This study aims to provide an improved reliability on the association between NBN polymorphisms and multiple cancers through the extended statistical meta-analysis. We collected a comprehensive dataset comprising 58 individual SNP-cancer association studies, including 23 494 cases and 29 592 controls for rs1805794, and 5325 cases and 11 149 controls for rs709816 polymorphisms, using a systematic search strategy across online databases. The collected data were analyzed using statistical meta-analysis to investigate the association between two SNPs and cancers. This meta-analysis revealed that the *C* allele of rs1805794 and rs709816 polymorphisms is not significantly associated with overall cancer risk in each ethnic population. However, sub-group analysis based on cancer types showed that rs1805794 is significantly associated with the increased risk of bladder cancer under three, and nasopharyngeal cancer (subtype of head and neck cancer) under four genetic models. Also, it was seen that rs1805794 is partially associated with brain cancer risk under allelic model, while rs709816 is significantly linked to breast cancer. Notably, rs1805794 exhibited a trend toward increased cancer risk, while rs709816 showed a protective tendency. Besides, bioinformatics analysis results also supported the meta-analysis results from different viewpoints including expression analysis of NBN gene from TCGA database, disease-gene and gene-regulator network analysis, and gene ontology and pathway enrichment analysis, and indicate the NBN gene directly/indirectly associated with cancer risks. Meta-analysis results, supported by bioinformatics analyses, indicate potential associations between NBN gene variants and susceptibility to bladder, brain, breast, and nasopharyngeal cancers. However, these findings are exploratory and indicate biological relevance rather than established diagnostic or prognostic utility. Systematic review registration: https://www.crd.york.ac.uk/prospero/; identifier: CRD420251034651.

## Introduction

Cancer is the second leading cause of death, accounting for ∼10 million deaths annually [1]. It develops due to the accumulation of genetic mutations that disrupt normal cellular functions. The NBN (Nibrin) gene, also known by aliases such as Nijmegen breakage syndrome 1 (NBS1), p95 protein of the MRE11/RAD50 complex, ATV, plays a crucial role in DNA repair and maintaining genome stability [2]. At least 10 mutations in the NBN gene have been linked to NBS, a condition characterized by growth retardation, recurrent infections, and an increased risk of cancer. Several genome-wide association studies (GWAS) or individual genetic association studies (IGAS) have explored the association between NBN gene single nucleotide polymorphisms (SNPs) and cancer risk across various cancer types using case-control samples. However, findings have been inconsistent, likely due to various cancer types, control group selection, ethnic diversity, and limited sample sizes [3–8]. To achieve a more accurate and comprehensive assessment, researchers have conducted statistical meta-analysis that integrate all available GWAS findings [3–13].

Over time, numerous individual GWAS or IGAS analyses have been conducted to investigate the association between the NBN gene polymorphisms (rs1805794 and rs709816) and multiple cancer types. Studies have examined this association in various cancers, including bladder cancer [14–20], blood cancer [21–30], bone cancer [31, 32], brain cancer [33, 34], breast cancer [35–49], colon cancer [48, 50–52], laryngeal cancer [20, 53, 54], liver cancer [55], lung cancer [20, 56–60], kidney [61], nasopharyngeal cancer [62, 63], ovarian cancer [64], prostate cancer [65], skin cancer [20, 66, 67], and thyroid cancer [68]. However, the reported associations have been inconsistent, varies across studies due to differences in case-control sample sizes, ethnic populations, and types of cancers. To establish a consensus on these associations across different cancer types and ethnic subgroups, meta-analysis is a crucial tool for evaluating the role of NBN polymorphisms as potential biomarkers. Previous meta-analyses reported that the SNP rs1805794 is significantly associated with blood and nasopharyngeal cancers [3], lung cancer [4, 8], and bladder cancer [4, 5], but showed no significant association with overall cancer risk [3, 4], particularly breast cancer [4, 7], as well as ovarian and colon cancers [4] and bone cancer [6]. Similarly, meta-analysis findings for rs709816 indicated no significant association with the bone cancer risk [6]. Meta-analysis relies on pooled estimates from fixed-effect or random-effect models, which are chosen based on homogeneity analysis. However, heterogeneity depends on the sample sizes within each cancer group and subgroups, necessitating updates to meta-data and statistical methods to enhance reliability. Although rs1805794 and rs709816 have been extensively investigated in earlier studies, inconsistencies in reported associations across cancer types remain unresolved. Moreover, several previous meta-analyses were limited by smaller sample sizes, restricted subgroup analyses, or a lack of integrative bioinformatic interpretation. Therefore, further investigations incorporating larger datasets and advanced analytical approaches remain essential for refining the understanding of NBN gene polymorphisms in cancer susceptibility.

In this context, the present study aims to provide an updated and comprehensive evaluation by incorporating a larger pooled dataset, refined subgroup and meta-regression analyses, and complementary functional insights. While the relative saturation of studies on these polymorphisms may limit the discovery of novel associations, this updated synthesis offers a more robust and reliable assessment of their role in cancer susceptibility.

In this study, we have conducted a comprehensive statistical meta-analysis to establish a consensus on the association between NBN gene polymorphisms (rs1805794, rs709816) and multiple cancer risks, utilizing a larger pooled dataset, refined subgroup and meta-regression analyses, and complementary functional insights. While the relative saturation of studies on these polymorphisms may limit the discovery of novel associations, this updated synthesis offers a more robust and reliable assessment of their role in cancer susceptibility. We systematically collected all available single studies related to these NBN gene polymorphisms and applied updated statistical methods. Importantly, the association of the NBN gene with multiple cancer risks was investigated not only at the SNP level but also across other platforms, including microarray, RNA-seq, and so on. Consequently, our approach integrated both statistical meta-analysis and bioinformatics tools to provide a robust evaluation of the NBN gene’s role in cancer susceptibility.

## Materials and methods

### Collection of meta-data related to NBN gene polymorphisms and multiple cancers

#### Literature search strategy

We conducted text-mined data searches up to December 2024, focusing exclusively on English-language literature across PubMed, PubMed Central, Google Scholar, and Web of Science. Our search employed a structured combination of keywords related to NBN gene polymorphisms, including individual SNP identifiers (“rs1805794” and “rs709816”), variations (“E185Q,” “Glu185Glu,” “8360G > C,” “1197 T > C”), and their combinations with cancer-specific terms (“rs1805794, cancer,” “rs709816, cancer,” “NBN, rs1805794, rs709816,” “NBS1, rs1805794, rs709816”). The initial screening involved reviewing titles and abstracts to select relevant studies, followed by full-text assessments conducted by two authors, with disagreements resolved through consensus revisions. Additionally, the reference lists of included studies were manually checked to identify further relevant publications. This comprehensive approach ensures the thoroughness of our review, reduces bias, and promotes transparency and reproducibility in accordance with best practices for systematic reviews.

### Eligibility criteria

We conducted the study based on the preferred reporting items for systematic reviews and meta-analyses (PRISMA) statements filed [69]. Also, this study was registered in PROSPERO (CRD420251034651). Initially, articles were screened and excluded based on their titles and abstracts if found irrelevant. Subsequently, studies were selected for the meta-analysis based on the following criteria: (i) the study investigated the association between NBN gene polymorphisms (rs1805794, rs709816) and cancer susceptibility, (ii) the study utilized a case-control or nested case-control design, and (iii) the study provided the necessary genotypic frequency information. Studies were excluded from the meta-analysis if they did not meet these specified criteria.

### Data extraction and quality assessment

Two authors independently investigated the eligible study to compile the datasets. We gathered essential information from each qualifying study including first author, year of publication, country, ethnicity (Asian, Caucasian, and Mixed), source of control populations (Hospital-based, Population-based, Mixed), number of cases and controls, type of cancers, and genotypic frequencies. To verify the accuracy of the included study, we applied the Hardy-Weinberg equilibrium (HWE) test based on the control populations using Chi-square statistic. A study was deemed suitable for inclusion in the meta-analysis if the HWE test yielded a *P* value ≥.05.

### Statistical meta-analysis

To accurately determine the association between the NBN gene polymorphisms (rs1805794, rs709816) and multiple cancer risks, we conducted a comprehensive statistical meta-analysis as follows. Initially, the accuracy of the studies included was verified using the HWE test, which was conducted using the Chi-square statistic. This test assessed the null hypothesis that the genotypic ratios under the control population were consistent, deeming a study suitable for meta-analysis if the *P* value was ≥.05.

We calculated the pooled odds ratio (OR) [70] and corresponding 95% confidence interval (CI) to assess the association between SNPs and cancer susceptibility. The pooled ORs were derived using either a fixed-effect (FE) or random-effect (RE) model, depending on the outcome of the heterogeneity analysis. The heterogeneity among the individual studies was evaluated by using Cochran’s Q-statistic [70, 71] and its extended Higgin’s and Thompson *I*^2^ statistic [70, 72]. Significant heterogeneity was indicated by a Q-test *P* value <.10 and an *I*^2^ value >50%. In cases of significant heterogeneity, the RE model was employed to estimate the pooled effect, otherwise, the FE model was utilized. The pooled ORs were calculated from genotypic case-control frequency datasets by using either the Mantel-Haenszel method [73] or the inverse variance method [74] for the FE or RE model, respectively.

The meta-data were analyzed using five different genetic models, such as (i) homozygote (CC vs. GG; CC vs. TT), (ii) recessive (CC vs. CG + GG; CC vs. CT + TT), (iii) dominant (CC + CG vs. GG; CC + CT vs. TT), (iv) heterozygote (CG vs. GG; CC vs. TT), and allelic contrast (C vs. G; C vs. T) models. The association between SNPs and cancer risk was visually represented in forest plots. Furthermore, subgroup analyses were conducted based on cancer types, ethnicity, and source of control populations to elucidate specific associations. To explore potential sources of heterogeneity, we conducted a meta-regression analysis using the RE model. Estimates were obtained using the restricted maximum likelihood (REML) method, and statistical significance was assessed with z-tests and 95% CI. The results were interpreted based on effect size estimates and their corresponding *P* values. Publication bias was assessed for each study both graphically, using funnel plots [75] and statistically, through Egger’s linear regression test [76] and Begg’s test [77]. These tests confirmed the absence of publication bias with *P* values >.05. Additionally, the sensitivity of meta-analysis results was tested by excluding studies that failed the HWE test. The methodological approach for conducting the meta-analysis is detailed extensively in the referenced study [10–13]. All the statistical analyses were performed using R-software (R × 64 3.5.2), supported by the “meta” package (http://meta-analysis-with-r.org/).

### Bioinformatics analysis

To support the link of NBN-gene polymorphisms with multiple cancers identified by statistical meta-analysis, we considered bioinformatics analysis by using different web-tools and databases. We performed expression analysis of NBN gene through Box plots with multiple cancers based on the Gene Expression Profiling Interactive Analysis 2 (GEPIA2) database that combined the cancer genome atlas (TCGA) and genotype-tissue expression (GTEx) databases [78], to investigate the differential expression patterns in cancerous and normal tissues. In order to further verify the link of NBN-gene with multiple cancers by integrating relevant data from different sources, we performed a disease-gene association study through the DisGeNET database constructed based on the data sources including curated, inferred, animal models and literature review [79]. To investigate the common pathogenetic processes of the NBN gene with some other cancer-causing genes/proteins (IL6, BRCA1, ADIPOQ, IGF1, ACYP2, TERT, PALB2) [10, 80–86], common transcriptional and post-transcriptional regulators were disclosed through the interaction network analysis of those genes including NBN with the transcription factors (TFs) and microRNAs by using the databases ENCODE and miRTarBase, respectively, in the NetworkAnalyst [87]. Then, we explored cancer-related gene ontology (GO) terms and KEGG pathways by the enrichment analysis of those genes using some popular bioinformatics web tools (GeneCodis [88], DEVID, Enrichr [89], and ToppFun [90]). Thus bioinformatics analysis results might support the link of NBN-gene with multiple cancers.

## Results

### Meta-analysis results

#### Study characteristics

**Figure 1:**
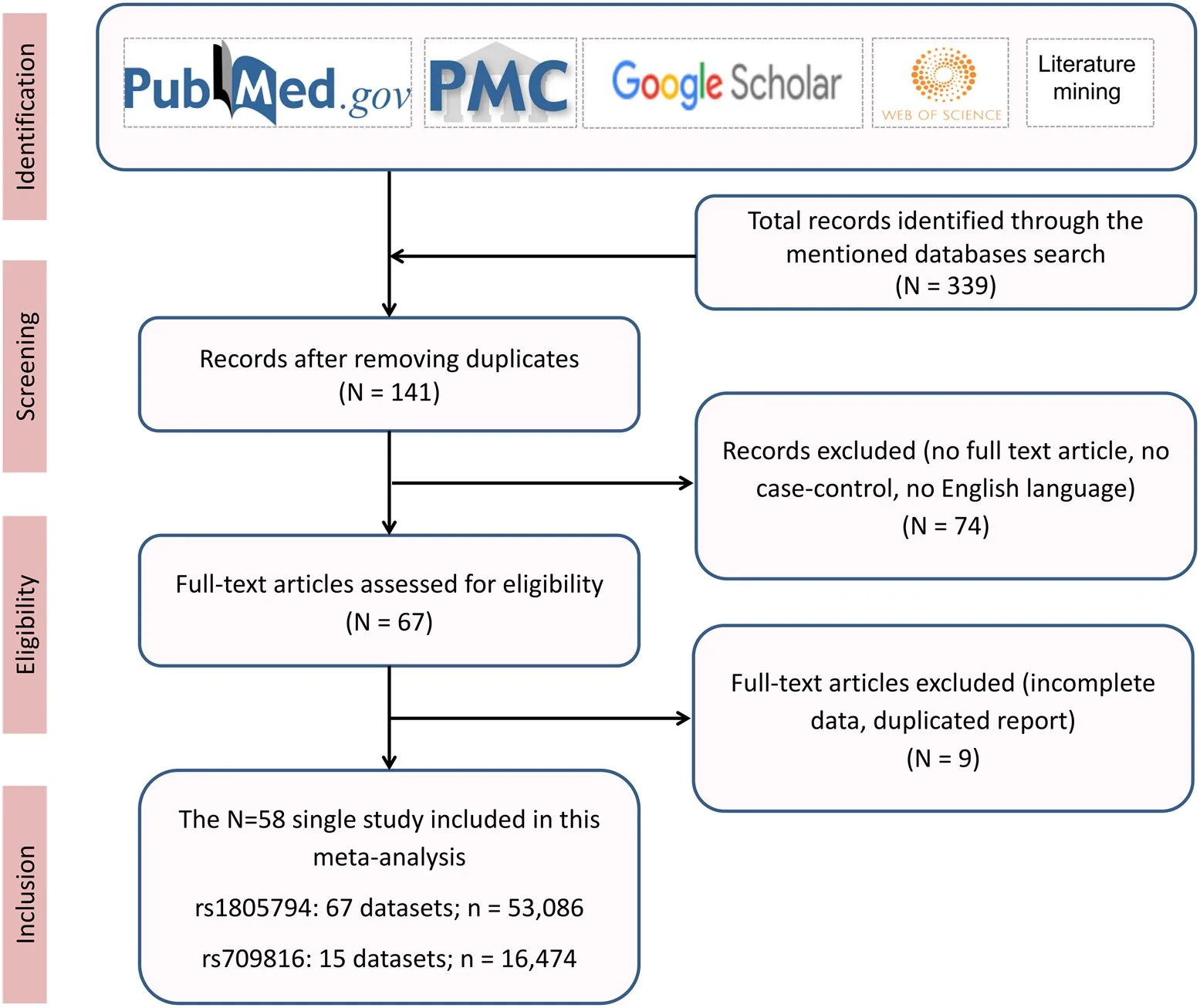
Systematic dataset construction process for meta-analysis (data searching, screening, selecting); where “*N*” represents the number of studies and “*n*” denotes the sample sizes.

Initially, we reviewed 339 articles retrieved through an online databases search. After removing duplicates, 141 studies remained. Further screening led to the selection of 58 individual studies, with 74 studies excluded due to incomplete information. The flow diagram of the study selection process is illustrated in Fig. 1. The selected 58 studies were categorized based on NBN gene polymorphisms, with 54 studies utilizing 67 datasets for rs1805794 and 11 studies utilizing 15 datasets for rs709816. The final dataset comprised a total of 53 086 subjects (cases: 23 494; controls: 29 592) for rs1805794 and 16 474 subjects (cases: 5325; controls: 11 149) for rs709816 polymorphisms. The subjects in these datasets represented 16 types of cancer, including bladder, blood, bone, brain, breast, colon, head and neck, kidney, laryngeal, liver, lung, nasopharyngeal, ovarian, prostate, skin, and thyroid cancers, across three ethnic groups (Asian, Caucasian, and mixed). For estimating the meta effect, cancer types with only one available study, such as head and neck, kidney, liver, and thyroid cancers for rs1805794, as well as bladder, head and neck, laryngeal, lung, ovarian, and skin cancers for rs709816 polymorphisms, were grouped and named as the “other” category. The “other” cancer category was used to represent the group of single studies, rather than implying biological similarity among them. This group is heterogeneous and should not be interpreted as a biologically unified cancer category, and findings from this subgroup have therefore been interpreted with caution. The detailed dataset information is provided in Table 1.

**Table 1:** Characteristic of included studies in this meta-analysis investigating the association of the NBN gene (rs1805794, rs709816) polymorphisms with multiple cancer types.^a^

Author	Year	Country	Ethnicity	Source of control	Case	Control	Cancer types	***P*_HWE_** ^b^
**rs1805794**								
** Kuschel *et al.* [49]**	2002	UK	Caucasian	Population	1034	864	Breast cancer	0.850 (Y)
** Kuschel *et al.* [49]**	2002	UK	Caucasian	Population	660	864	Breast cancer	0.850 (Y)
** Sanyal *et al.* [19]**	2004	Sweden	Caucasian	Hospital	299	278	Bladder cancer	0.234 (Y)
** Forsti *et al.* [38]**	2004	Germany	Caucasian	Population	221	319	Breast cancer	0.920 (Y)
** Broberg *et al.* [17]**	2005	Sweden	Caucasian	Hospital	61	154	Bladder cancer	0.383 (Y)
** Auranen *et al.* [64]**	2005	UK	Caucasian	Population	721	848	Ovarian cancer	0.381 (Y)
** Auranen *et al.* [64]**	2005	USA	Caucasian	Population	308	383	Ovarian cancer	0.455 (Y)
** Auranen *et al.* [64]**	2005	Denmark	Caucasian	Population	299	827	Ovarian cancer	0.948 (Y)
** Auranen *et al.* [64]**	2005	UK	Caucasian	Population	258	734	Ovarian cancer	0.850 (Y)
** Millikan *et al.* [46]**	2005	USA	Caucasian	Population	1273	1136	Breast cancer	0.660 (Y)
** Millikan *et al.* [46]**	2005	USA	Mixed	Population	766	681	Breast cancer	0.533 (Y)
** Festa *et al.* [66]**	2005	Sweden	Caucasian	Hospital	241	574	Skin cancer	0.937 (Y)
** Zhang *et al.* [37]**	2005	China	Asian	Hospital	220	310	Breast cancer	0.755 (Y)
** Lan *et al.* [59]**	2005	China	Asian	Population	118	111	Lung cancer	0.319 (Y)
** Wu *et al.* [16]**	2006	USA	Caucasian	Population	604	595	Bladder cancer	0.618 (Y)
** Shen *et al.* [23]**	2006	USA	Mixed	Population	477	563	Blood cancer	0.365 (Y)
** Lu *et al.* [47]**	2006	China	Caucasian	Hospital	421	423	Breast cancer	0.000 (N)
** Thirumaran *et al.* [67]**	2006	Germany	Caucasian	Hospital	529	533	Skin cancer	0.222 (Y)
** Zienolddiny *et al.* [58]**	2006	Norway	Caucasian	Hospital	310	376	Lung cancer	0.495 (Y)
** Hebbring *et al.* [65]**	2006	USA	Mixed	Population	200	200	Prostate cancer	0.047 (N)
** Hebbring *et al.* [65]**	2006	USA	Mixed	Population	121	200	Prostate cancer	0.047 (N)
** Ryk *et al.* [60]**	2006	Sweden	Caucasian	Population	177	152	Lung cancer	0.000 (N)
** Tarasov *et al.* [39]**	2006	Russia	Caucasian	Population	151	191	Breast cancer	0.982 (Y)
** Figueroa *et al.* [18]**	2007	USA	Caucasian	Hospital	1086	1020	Bladder cancer	0.117 (Y)
** Smith *et al.* [35]**	2008	USA	Caucasian	Hospital	318	407	Breast cancer	0.522 (Y)
** Smith *et al.* [35]**	2008	USA	Mixed	Hospital	53	74	Breast cancer	0.169 (Y)
** Choudhury *et al.* [15]**	2008	UK	Mixed	Mixed	748	788	Bladder cancer	0.414 (Y)
** Pardini *et al.* [50]**	2008	Czech	Caucasian	Hospital	532	532	Colon cancer	0.076 (Y)
** Mosor *et al.* [28]**	2008	Poland	Caucasian	Population	157	275	Blood cancer	0.266 (Y)
** Ziolkowska *et al.* [54]**	2008	Poland	Caucasian	Hospital	184	195	Laryngeal cancer	0.393 (Y)
** Margulis *et al.* [61]**	2009	USA	Caucasian	Hospital	322	333	Kidney cancer	0.012 (N)
** Maria *et al.* [43]**	2009	Cyprus	Asian	Population	1104	1154	Breast cancer	0.650 (Y)
** Bastos *et al.* [68]**	2009	Portugal	Caucasian	Hospital	109	217	Thyroid cancer	0.178 (Y)
** Desjardins *et al.* [45]**	2009	Canada	Caucasian	Population	97	73	Breast cancer	0.608 (Y)
** Schuetz *et al.* [29]**	2009	Canada	Caucasian	Population	160	582	Blood cancer	0.235 (Y)
** Schuetz *et al.* [29]**	2009	Canada	Caucasian	Population	168	582	Blood cancer	0.235 (Y)
** Schuetz *et al.* [29]**	2009	Canada	Caucasian	Population	67	582	Blood cancer	0.235 (Y)
** Rajaraman *et al.* [33]**	2010	USA	Caucasian	Hospital	527	469	Brain cancer	0.406 (Y)
** Fan *et al.* [56]**	2010	China	Asian	Hospital	254	488	Lung cancer	0.312 (Y)
** Silva *et al.* [42]**	2010	Portugal	Caucasian	Hospital	289	548	Breast cancer	0.365 (Y)
** Jelonek *et al.* [48]**	2010	Poland	Caucasian	Population	132	153	Colon cancer	0.893 (Y)
** Jelonek *et al.* [48]**	2010	Poland	Caucasian	Population	104	110	Head and neck cancer	0.446 (Y)
** Jelonek *et al.* [48]**	2010	Poland	Caucasian	Population	93	425	Breast cancer	0.411 (Y)
** Betti *et al.* [57]**	2011	Italy	Caucasian	Population	133	252	Lung cancer	0.757 (Y)
** Zheng *et al.* [63]**	2011	China	Asian	Hospital	700	758	Nasopharyngeal cancer	0.949 (Y)
** Zheng *et al.* [63]**	2011	China	Asian	Hospital	352	410	Nasopharyngeal cancer	0.346 (Y)
** Jiang *et al.* [24]**	2011	China	Asian	Hospital	175	350	Blood cancer	0.154 (Y)
** Jiao *et al.* [22]**	2012	China	Mixed	Population	150	141	Blood cancer	0.000 (N)
** Jiao *et al.* [22]**	2012	China	Mixed	Population	327	422	Blood cancer	0.000 (N)
** Huang *et al.* [55]**	2012	China	Asian	Hospital	865	900	Liver cancer	0.251 (Y)
** Erculj *et al.* [26]**	2012	Slovenia	Caucasian	Hospital	20	39	Blood cancer	0.340 (Y)
** Gil *et al.* [52]**	2012	Poland	Caucasian	Hospital	133	100	Colon cancer	0.627 (Y)
** Mosor *et al.* [21]**	2013	Poland	Caucasian	Hospital	232	275	Blood cancer	0.266 (Y)
** Wu *et al.* [36]**	2013	USA	Mixed	Population	329	397	Breast cancer	0.197 (Y)
** Li *et al.* [27]**	2013	China	Asian	Population	428	600	Blood cancer	0.764 (Y)
** Smolkova *et al.* [25]**	2014	Germany	Caucasian	Population	460	545	Blood cancer	0.607 (Y)
** Jin *et al.* [31]**	2015	China	Asian	Hospital	148	298	Bone cancer	0.000 (N)
** Zhang *et al.* [34]**	2015	China	Asian	Hospital	604	665	Brain cancer	0.000 (N)
** Li *et al.* [51]**	2015	China	Asian	Hospital	763	892	Colon cancer	0.398 (Y)
** Goricar *et al.* [32]**	2015	Slovenia	Caucasian	Hospital	79	373	Bone cancer	0.000 (N)
** Uzunoglu *et al.* [44]**	2016	Turkey	Caucasian	Population	101	115	Breast cancer	0.147 (Y)
** Hu *et al.* [53]**	2018	China	Asian	Hospital	342	345	Laryngeal cancer	0.081 (Y)
** Psoma *et al.* [62]**	2019	Greece	Caucasian	Hospital	17	57	Nasopharyngeal cancer	0.112 (Y)
** Chen *et al.* [14]**	2020	China	Asian	Mixed	763	892	Bladder cancer	0.398 (Y)
** Dimitrakopoulos *et al.* [91]**	2021	Greece	Caucasian	Hospital	142	149	Nasopharyngeal cancer	0.707 (Y)
** Zehtab *et al.* [92]**	2022	Iran	Asian	Hospital	50	50	Blood cancer	0.046 (N)
** Minina *et al.* [93]**	2022	Russia	Caucasian	Population	208	244	Lung cancer	0.073 (Y)
**rs709816**								
** Auranen *et al.* [64]**	2005	Turkey	Caucasian	Mixed	1609	3899	Ovarian cancer	0.117 (Y)
** Rollinson *et al.* [30]**	2006	UK	Caucasian	Population	442	445	Blood cancer	0.935 (Y)
** Lu *et al.* [47]**	2006	China	Caucasian	Hospital	421	423	Breast cancer	0.515 (Y)
** Hsu *et al.* [41]**	2007	China	Asian	Hospital	559	1125	Breast cancer	0.055 (Y)
** Sehl *et al.* [40]**	2009	USA	Caucasian	Population	196	203	Breast cancer	0.000 (N)
** Park *et al.* [20]**	2010	USA	Mixed	Mixed	513	893	Lung cancer	0.146 (Y)
** Park *et al.* [20]**	2010	USA	Mixed	Mixed	381	902	Skin cancer	0.110 (Y)
** Park *et al.* [20]**	2010	USA	Mixed	Mixed	225	902	Head and Neck cancer	0.110 (Y)
** Park *et al.* [20]**	2010	USA	Mixed	Mixed	148	150	Bladder cancer	0.024 (N)
** Park *et al.* [20]**	2010	USA	Mixed	Mixed	76	902	Laryngeal cancer	0.110 (Y)
** Erculj *et al.* [26]**	2012	Slovenia	Caucasian	Hospital	20	39	Blood cancer	0.465 (Y)
** Smolkova *et al.* [25]**	2014	Germany	Caucasian	Population	458	545	Blood cancer	0.659 (Y)
** Jin *et al.* [31]**	2015	China	Asian	Hospital	148	298	Bone cancer	0.000 (N)
** Goricar *et al.* [32]**	2018	Slovenia	Caucasian	Hospital	79	373	Bone cancer	0.000 (Y)
** Zehtab *et al.* [92]**	2022	Iran	Asian	Hospital	50	50	Blood cancer	0.637 (Y)

a All the included studies are arranged by ascending order of the publication year.

b HWE, Hardy-Weinberg equilibrium; *P*, probability value; Y and N, passed and failed on HWE test, respectively.

### Quantitative synthesis of rs1805794 (G > C)

**Figure 2:**
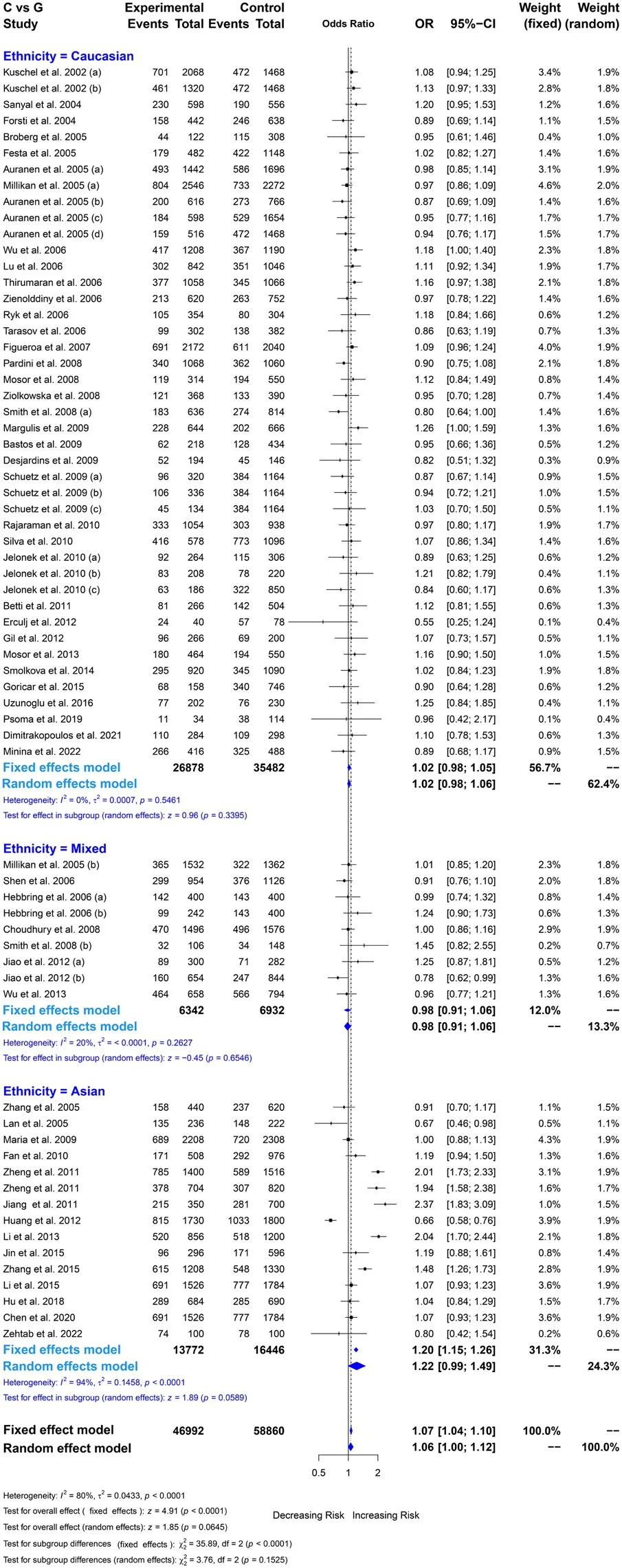
The association between rs1805794 polymorphism and cancer risk by showing the Forest plot under allelic comparison (C vs. G) with different ethnic populations. The square represents the ORs with their weighted size, the parallel lines indicate 95% CI, and the diamond plot indicates the overall or estimate.

We performed a statistical meta-analysis for the rs1805794 SNP, which showed the insignificant association between NBN gene rs1805794 polymorphism and overall cancer risk under the all genetic models (CC vs. GG*:* OR =1.06, 95% CI = 1.00–1.12, *P = .*0645; CC vs. CG + GG: OR = 1.13, 95% CI =0.99–1.29, *P = .*0748; CC + CG vs. GG: OR =1.05, 95% CI = 0.98–1.11, *P = .*1631; CG vs. GG: OR =1.07, 95% CI = 0.99–1.19, *P = .*0862; C vs. G: OR = 1.10, 95% CI = 0.98–1.23, *P = .*0993; Table 2 and Fig. 2, Figs. S1–S4). Although the association with overall cancer risk was statistically insignificant, all the ORs were greater than the null value (OR = 1), suggesting statistical significance was not achieved, and the null hypothesis cannot be rejected. Subgroup analysis by cancer types revealed a significant association with increasing the risk between rs1805794 and bladder cancer under three genetic models (CC vs. GG: OR = 1.09, *P = .*0193; CC + CG vs. GG: OR = 1.13, *P = .*0186; CG vs. GG: OR = 1.13, *P = .*0110), and with nasopharyngeal cancer under all genetic models (CC vs. GG: OR = 1.56, *P = .*0120; CC vs. CG + GG: OR = 2.28, *P = .*0342; CC + CG vs. GG: OR = 1.58, *P = .*0001; CG vs. GG: OR = 1.98, *P = .*0001). However, this polymorphism showed an insignificant association with other cancer types under any genetic models (Table 2). Subgroup analysis by ethnicity indicated that rs1805794 polymorphism was not significantly associated with overall cancer risk in Asian, Caucasian, or mixed populations under any genetic models (Table 2). When analyzed based on the source of the control population, the hospital-based subgroup showed a significant association with overall cancer risk under two genetic models (CC vs. GG: OR = 1.11, *P = .*0461; CC vs. CG + GG: OR = 1.27, 0.0410), whereas no significant association was observed for population-based controls (Table 2).

**Table 2:** The summary associations between the NBN gene rs1805794 and rs709816 polymorphisms and cancer risk.^a^

rs1805794 (G > C)	Study number	Sample size	CC vs. GG		CC vs. CG + GG		CC + CG vs. GG		CG vs. GG		C vs. G	
			OR [95% CI]	*P* value	OR [95% CI]	*P* value	OR [95% CI]	*P* value	OR [95% CI]	*P* value	OR [95% CI]	*P* value
**Overall**	67	53 086	1.06 [1.00–1.12]	.0645	1.13 [0.99–1.29]	.0784	1.05 [0.98–1.11]	.1631	1.07 [0.99–1.15]	.0862	1.1 [0.98–1.23]	.0993
**HWE-yes**	56	47 230	1.05 [0.98–1.12]	.1673	1.09 [0.95–1.26]	.2188	1.04 [0.97–1.11]	.2831	1.05 [0.97–1.14]	.200	1.08 [0.96–1.21]	.210
**Breast cancer**	16	15 111	1.00 [0.95–1.05]	.8995	0.99 [0.89–1.11]	.9126	1.01 [0.94–1.09]	.6942	1.01 [0.94–1.08]	.7773	0.99 [0.9–1.1]	.8712
**Bladder cancer**	6	7288	**1.09 [1.01**–**1.16]**	**.0193**	1.15 [0.98–1.33]	.0794	**1.13 [1.02**–**1.24]**	**.0186**	**1.13 [1.03**–**1.24]**	**.011**	1.07 [0.93–1.24]	.3216
**Ovarian cancer**	4	4378	0.95 [0.86–1.04]	.2454	0.85 [0.69–1.05]	.1326	1.0 [0.87–1.14]	.9645	0.97 [0.85–1.1]	.5842	0.85 [0.69–1.04]	.1123
**Skin cancer**	2	1877	1.10 [0.96–1.26]	.1852	1.2 [0.9–1.61]	.2209	1.09 [0.89–1.33]	.4169	1.11 [0.92–1.35]	.2653	1.15 [0.88–1.52]	.3106
**Lung cancer**	6	2823	1.01 [0.9–1.13]	.8595	0.88 [0.55–1.41]	.5945	1.05 [0.88–1.25]	.5748	1.05 [0.89–1.24]	.556	0.96 [0.67–1.38]	.8192
**Blood cancer**	13	7877	1.1 [0.9–1.35]	.3458	1.47 [0.93–2.33]	.0981	1.03 [0.81–1.3]	.8288	1.11 [0.84–1.47]	.4478	1.34 [0.95–1.88]	.0992
**Prostate cancer**	2	721	1.09 [0.88–1.36]	.4201	0.98 [0.62–1.55]	.9351	1.48 [1.07–2.04]	.0179	1.33 [0.98–1.8]	.0634	0.8 [0.53–1.22]	.3029
**Colon cancer**	4	3237	1.00 [0.9–1.11]	.9932	0.95 [0.77–1.18]	.6491	1.09 [0.94–1.27]	.2646	1.05 [0.91–1.22]	.4994	0.91 [0.75–1.11]	.3615
**Laryngeal cancer**	2	1066	1.01 [0.85–1.2]	.9332	1.12 [0.76–1.64]	.5691	0.9 [0.69–1.18]	.4495	0.94 [0.73–1.22]	.6572	1.15 [0.81–1.62]	.4379
**Brain cancer**	2	2265	1.2 [0.79–1.82]	.3885	1.5 [0.91–2.47]	.1079	1.06 [0.62–1.81]	.8247	1.17 [0.65–2.11]	.5985	**1.49 [1.2**–**1.84]**	**.0003**
**Nasopharyngeal cancer**	4	2585	**1.56 [1.1**–**2.2]**	**.012**	**2.28 [1.06**–**4.89]**	**.0342**	**1.58 [1.32**–**1.9]**	**.0001**	**1.98 [1.66**–**2.35]**	**.0001**	1.85 [0.93–3.69]	.0800
**Bone cancer**	2	898	1.06 [0.84–1.33]	.6346	2.65 [0.16–4.47]	.4941	0.96 [0.43–2.14]	.9225	0.96 [0.43–2.14]	.9225	2.94 [0.18–4.86]	.4488
**Others**	4	2960	0.97 [0.7–1.33]	.8463	1.03 [0.46–2.29]	.9417	0.85 [0.62–1.16]	.3000	0.88 [0.59–1.31]	.5198	1.13 [0.59–2.19]	.7121
**Ethnicity**												
** Asian**	15	15 109	1.22 [0.99–1.49]	.0589	1.53 [0.97–2.41]	.0683	1.2 [1.0–1.45]	.055	1.29 [0.99–1.68]	.0551	1.34 [0.97–1.84]	.0726
** Caucasian**	43	31 340	1.02 [0.98–1.05]	.3047	1.05 [0.97–1.14]	.2169	1.0 [0.95–1.05]	.9076	1.01 [0.97–1.06]	.5953	1.05 [0.95–1.16]	.3079
** Mixed**	9	6637	0.98 [0.91–1.06]	.6483	0.93 [0.77–1.12]	.4292	1.1 [0.9–1.35]	.3654	1.06 [0.88–1.26]	.5513	0.9 [0.77–1.06]	.2051
**Source of controls**												
** PB**	33	26 926	1.01 [0.95–1.09]	.713	1.02 [0.87–1.19]	.8149	1.02 [0.94–1.1]	.6443	1.02 [0.93–1.12]	.6755	1.02 [0.9–1.15]	.8110
** HB**	32	22 969	**1.11 [1.0**–**1.23]**	**.0461**	**1.27 [1.01**–**1.6]**	**.0410**	1.07 [0.97–1.19]	.1787	1.12 [0.99–1.28]	.0679	1.2 [0.99–1.45]	.0695
** Mixed**	2	3191	1.04 [0.94–1.15]	.4725	1.04 [0.83–1.29]	.7409	1.11 [0.95–1.29]	.1955	1.09 [0.94–1.26]	.2576	0.98 [0.81–1.2]	.8805

rs709816 (T > C)	Study number	Sample size	CC vs. TT		CC vs. CT + TT		CC + CT vs. TT		CT vs. TT		C vs. T	
			OR [95% CI]	*P* value	OR [95% CI]	*P* value	OR [95% CI]	*P* value	OR [95% CI]	*P* value	OR [95% CI]	*P* value
**Overall**	15	16 474	**0.95 [0.9**–**1.00]**	**.0469**	0.91 [0.82–1.01]	.0709	0.97 [0.88–1.07]	.5335	0.94 [0.86–1.04]	.221	0.93 [0.87–1.00]	.056
**HWE-Yes**	12	15 331	0.96 [0.91–1.01]	.0831	0.91 [0.82–1.02]	.0982	0.96 [0.87–1.06]	.4470	0.94 [0.85–1.04]	.217	0.94 [0.87–1.02]	.124
**Blood cancer**	4	2049	0.96 [0.85–1.09]	.561	0.93 [0.7–1.22]	.590	0.96 [0.74–1.26]	.7916	0.95 [0.73–1.22]	.674	0.95 [0.80–1.14]	.606
**Breast cancer**	3	898	**0.88 [0.77**–**0.99]**	**.0362**	0.65 [0.33–1.27]	.2054	0.55 [0.24–1.26]	.1588	0.65 [0.34–1.26]	.203	0.90 [0.75–1.07]	.231
**Bone cancer**	2	2927	0.99 [0.79–1.25]	.9358	1.01 [0.61–1.67]	.9618	1.39 [0.1–22.55]	.8178	1.01 [0.61–1.67]	.960	0.97 [0.71–1.33]	.866
**Others**	6	10 600	0.96 [0.91–1.03]	.2623	0.95 [0.83–1.07]	.3875	1.03 [0.91–1.16]	.6615	0.99 [0.88–1.11]	.880	0.93 [0.85–1.02]	.133
**Asian**	3	2230	0.92 [0.81–1.05]	.2051	0.82 [0.62–1.09]	.1771	0.86 [0.67–1.1]	.2237	0.84 [0.66–1.07]	.158	0.92 [0.76–1.12]	.411
**Caucasian**	7	9152	0.97 [0.91–1.04]	.3954	0.95 [0.82–1.09]	.4447	0.97 [0.84–1.12]	.6618	0.96 [0.84–1.1]	.552	0.97 [0.88–1.06]	.454
**Mixed**	5	5092	0.93 [0.85–1.02]	.1142	0.88 [0.74–1.06]	.1865	1.02 [0.87–1.21]	.7855	0.97 [0.83–1.13]	.663	**0.87 [0.75**–**1.00]**	**.044**
**PB**	3	2289	0.97 [0.85–1.1]	.5878	0.92 [0.71–1.2]	.5451	0.91 [0.69–1.2]	.5168	0.92 [0.72–1.18]	.509	0.98 [0.82–1.16]	.781
**HB**	6	3585	0.9 [0.81–1.00]	.0544	**0.79 [0.62**–**1.00]**	**.0475**	0.8 [0.63–1.02]	.0736	0.82 [0.67–1.00]	.056	0.90 [0.77–1.05]	.181
**Mixed**	6	10 600	0.96 [0.91–1.03]	.2623	0.95 [0.83–1.07]	.3875	1.03 [0.91–1.16]	.6615	0.99 [0.88–1.11]	.880	0.93 [0.85–1.02]	.133

a The bold results indicate statistical significance. The ORs of ethnicity and source of control population subgroups represent the overall cancer risk.

### Quantitative synthesis of rs709816 (T > C)

**Figure 3:**
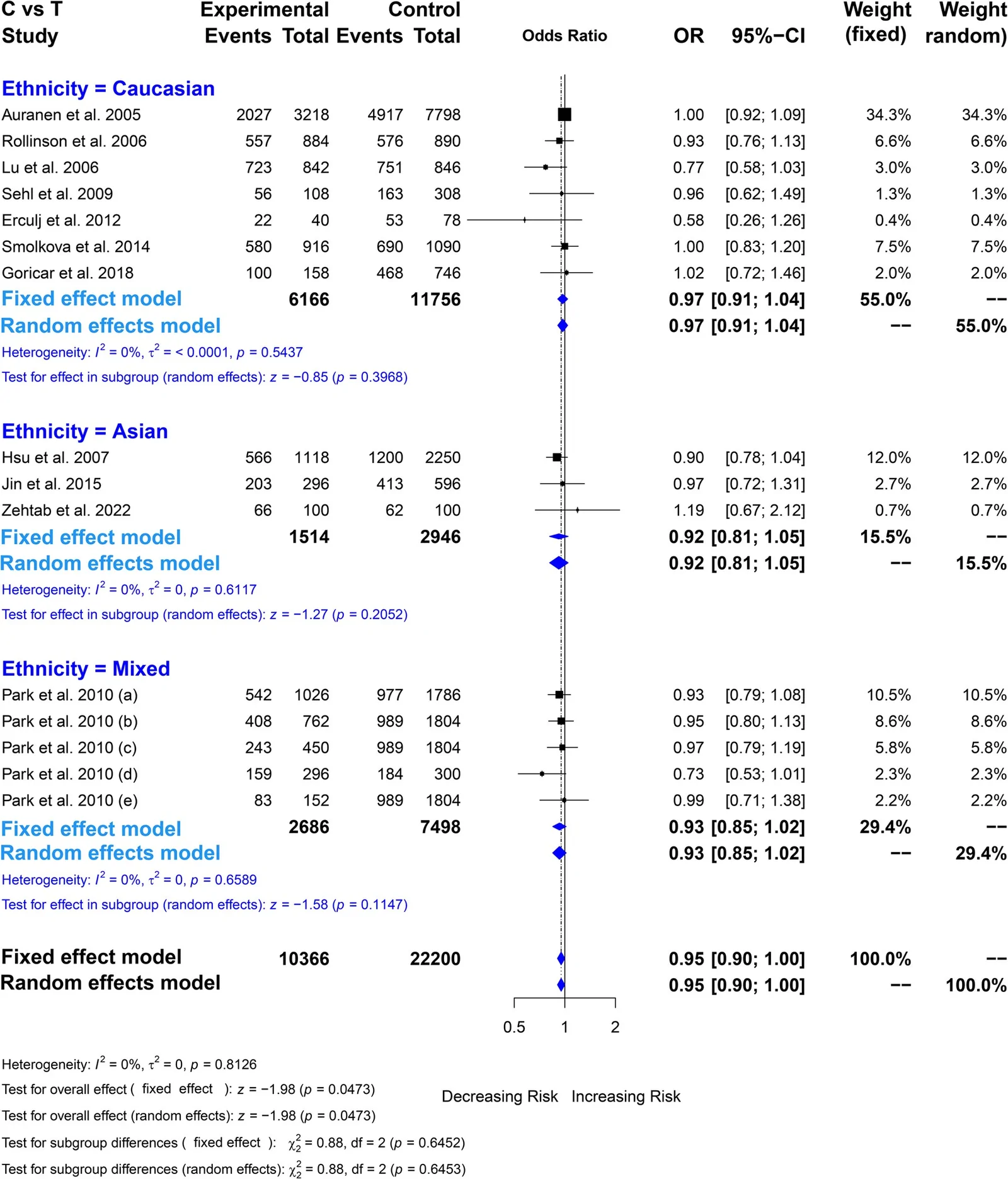
The association between rs709816 polymorphism and cancer risk by showing the Forest plot under allelic comparison (C vs. T) with different ethnic populations.

The meta-analysis results indicated a significant association between NBN gene rs709816 polymorphism and overall cancer risk under a single genetic model (CC vs. TT: OR = 0.95, *P = .*0469) (see Table 2, Fig. 3, Figs. S5–S8). The subgroup analysis by cancer types revealed a significant association with decreasing the risk between rs709816 and breast cancer under a single genetic model (CC vs. TT: OR = 0.88, *P = .*0362). However, this polymorphism showed an insignificant association with the rest of the specific cancer subgroups. Subgroup analysis by ethnicity revealed no significant association between rs709816 and overall cancer in any population, except for the mixed population under the allelic model (C vs. T: OR = 0.87, *P = .*0440; Table 2). When analyzed based on the source of the control population, a significant association was observed in the hospital-based population under the recessive model (CC vs. CT + TT), while no significant association was found in population-based controls (Table 2).

### Source of heterogeneity

We observed the significant heterogeneity of NBN gene rs1805794 polymorphism for overall cancer risk through all the genetic models (CC vs. GG: Q = 316.04, *df* = 66, *P = .*0001, *I*^2^ = 79.1%; CC+CG vs. GG: Q = 218.77, *df* = 66, *P = .*0001, *I*^2^ = 69.8%; CG vs. GG: Q = 139.33, *df* = 66, *P = .*0001, *I*^2^ = 52.6%; C vs. G: Q = 328.03, *df* = 66, *P = .*0001, *I*^2^ = 79.9%; Table S1). The subgroup of blood, brain, nasopharyngeal, and other cancers with the ethnicity of Asian and Caucasian, as well as HB and PB control populations, showed the main sources of heterogeneity. To specify the potential sources of heterogeneity across various genetic models (C vs. G, CC vs. GG, CG vs. GG, CC + CG vs. GG, CC vs. CG + GG), we conducted a meta-regression analysis within the subgroup characterized by high *I*^2^ (>50%) values. The results for the overall cancer group indicated that ethnicity, cancer type, and case-control ratio significantly contributed to heterogeneity in specific models (Table S2, Fig. S9). Among cancer types, liver cancer demonstrated a significant negative association with effect size across multiple models, particularly in the C vs. G (*P < .*001), CC vs. GG (*P < .*001), CG vs. GG (*P < .*001), CC + CG vs. GG (*P < .*001), and CC vs. CG + GG (*P = .*012) models. This suggests that genetic effects in rs1805794 may vary depending on the cancer type (Table S2, Fig. S9). Ethnicity (Caucasian and mixed populations) also appeared as a key factor influencing effect size across several models, with significant associations in the C vs. G, CC vs. GG, CG vs. GG, and CC + CG vs. GG models (*P < .*05 for all comparisons) (Table S2, Fig. S9). In subgroup analyses, ethnicity remained a significant moderator in the blood cancer and HB subgroups, while the case-control ratio was a significant factor in the Asian and blood cancer subgroups (*P < .*05). No significant moderators were identified in the PB subgroup (Table S2, Fig. S9). The NBN gene rs709816 polymorphism did not exhibit significant heterogeneity for overall cancer risk or across all subgroups, which may be attributed to the small number of individual studies included in the analysis. All the heterogeneity results are shown in Tables S1 and S2.

### Publication bias

**Figure 4:**
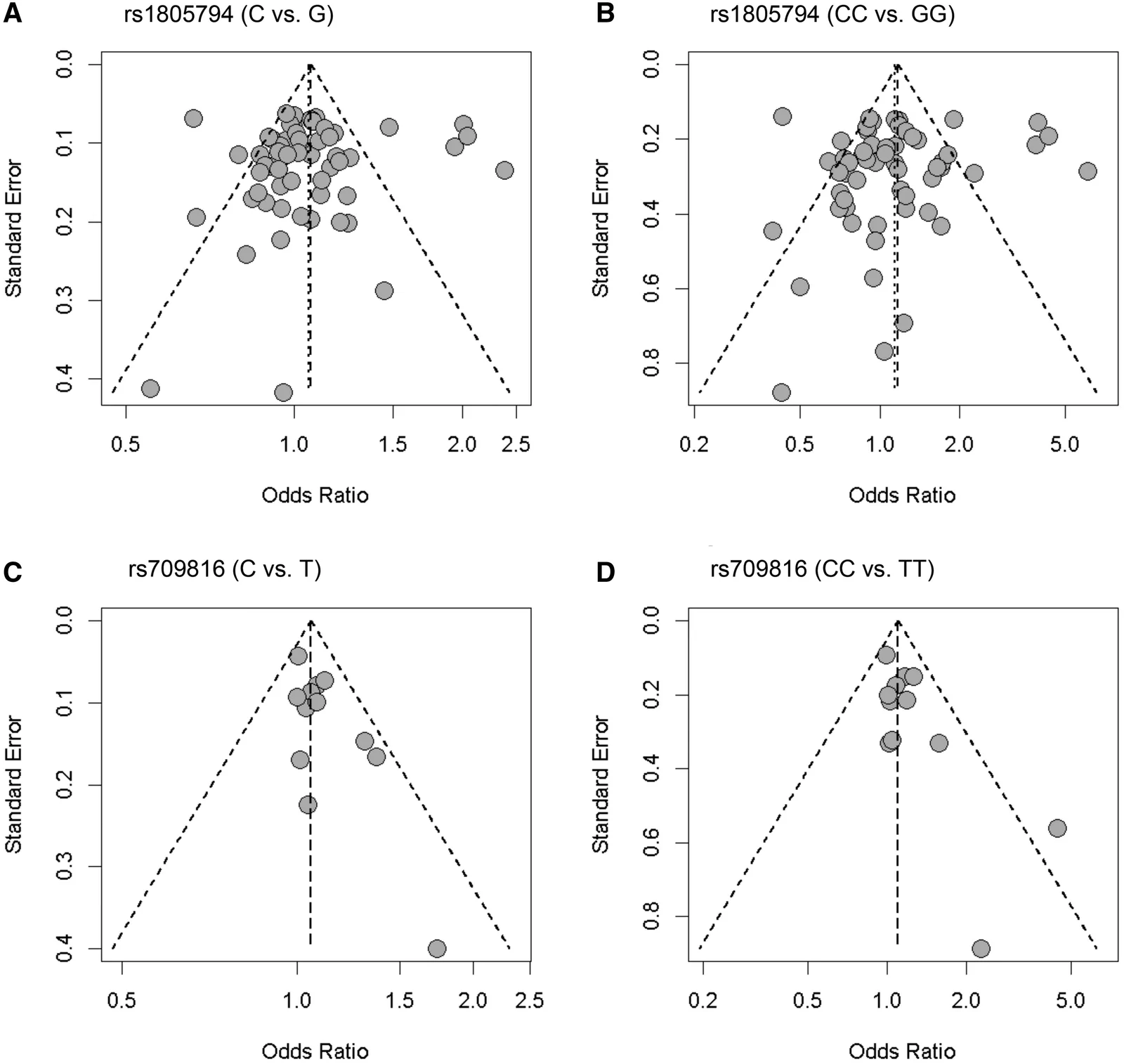
Funnel plots assessing publication bias in studies examining polymorphisms in the NBN gene. Each plot uses the standard error to evaluate the asymmetry in the distribution of odds ratios from individual studies, indicating the absence of publication bias. Panel (A) shows rs1805794 for the C vs. G allele comparison, (B) for the CC vs. GG genotype comparison, (C) for rs709816 C vs. T allele comparison, and (D) for the CC vs. TT genotype comparison. Each circle represents an individual study, and the dashed lines indicate the expected distribution under no publication bias.

The publication bias of the eligible studies was assessed visually using funnel plots (Fig. 4) for the rs1805794 and rs709816 polymorphisms under two respective genetic combinations (C vs. G; CC vs. GG; C vs. T; CC vs. TT). The symmetry of funnel plots indicated no apparent publication bias. Further statistical validation using Begg’s test (C vs. G: *P = .*8342; CC vs. GG: *P = .*9374; C vs. T: *P = .*4106; CC vs. TT: *P = .*2171; Table S3) confirmed the absence of publication bias for both polymorphisms. However, Egger’s test (C vs. G: *P = .*6494; CC vs. GG: *P = .*5127; C vs. T: *P = .*0211; CC vs. TT: *P = .*0223; Table S3) suggested that while rs1805794 showed no publication bias, rs709816 exhibited bias under the allelic (C vs. T) and homozygote (CC vs. TT) models.

### Sensitivity analysis

We conducted a sensitivity analysis to assess robustness of our findings and the reliability of this meta-analysis. The overall cancer risk was evaluated using two datasets: (i) including all eligible studies and (ii) excluding studies that did not confirm HWE testing (Table 2). Although the overall estimates remained largely consistent between the two datasets, several associations were modest in magnitude and in some cases approached borderline statistical significance, suggesting that the observed effects may be relatively weak. Furthermore, potential publication bias, particularly for rs709816, as indicated by Egger’s test, may have influenced these results. Therefore, while the sensitivity analysis supports the overall stability of the findings, the associations should be interpreted cautiously and considered exploratory rather than definitive.

### False positive report probability (FPRP) and power analyses

To evaluate the reliability of the meta-analysis findings and assess the likelihood of false-positive associations, we conducted a false-positive report probability (FPRP) analysis. In the experiment, the biological importance parameter using a prior probability (π) of 0.01, and the FPRP threshold was 0. 2 [94]. An OR of 1.5 was considered for increasing risk, while an OR of 0.67 was used for a protective effect, allowing us to estimate the statistical power and FPRP [95]. Previous studies suggest that an OR of 1.5 and an FPRP of 0.2 serve as plausible thresholds for detecting a biologically significant effect [96–98]. The results of our analysis confirmed that the rs1805794 SNP was significantly associated with an increased risk of nasopharyngeal and brain cancers (Table S4).

### Bioinformatics results

**Figure 5:**
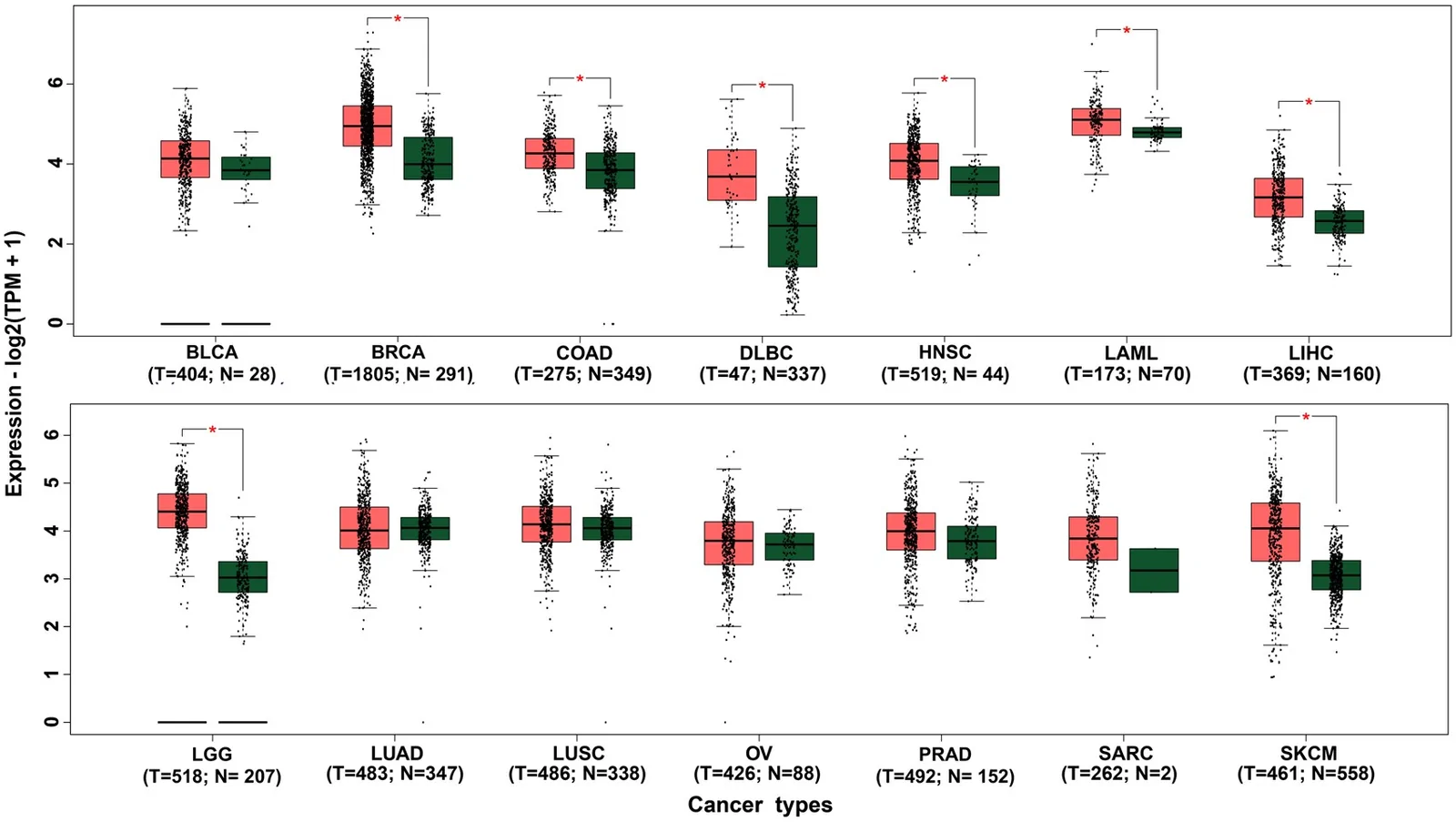
Differential expression of the NBN gene across multiple cancer types compared to control groups. Rectangle boxes denote the median and interquartile ranges of expression levels in cancer and control samples. Data were analyzed and visualized using the GEPIA-2 web server, with a log2 fold change cutoff of 0.5 and a significance level (*P*-value) of .05. Asterisks (*) above the boxes signify statistically significant differences from control groups. Figure represents different cancer types, including bladder urothelial carcinoma (BLCA), breast invasive carcinoma (BRCA), colon adenocarcinoma (COAD), diffuse large B-cell lymphoma (DLBC), head and neck squamous cell carcinoma (HNSC), acute myeloid leukemia (LAML), liver hepatocellular carcinoma (LIHC), lower grade glioma (LGG), lung adenocarcinoma (LUAD), lung squamous cell carcinoma (LUSC), ovarian serous cystadenocarcinoma (OV), prostate adenocarcinoma (PRAD), sarcoma (SARC), and skin cutaneous melanoma (SKCM). tumor (T) and normal (N) sample sizes are indicated below each cancer type.

**Figure 6:**
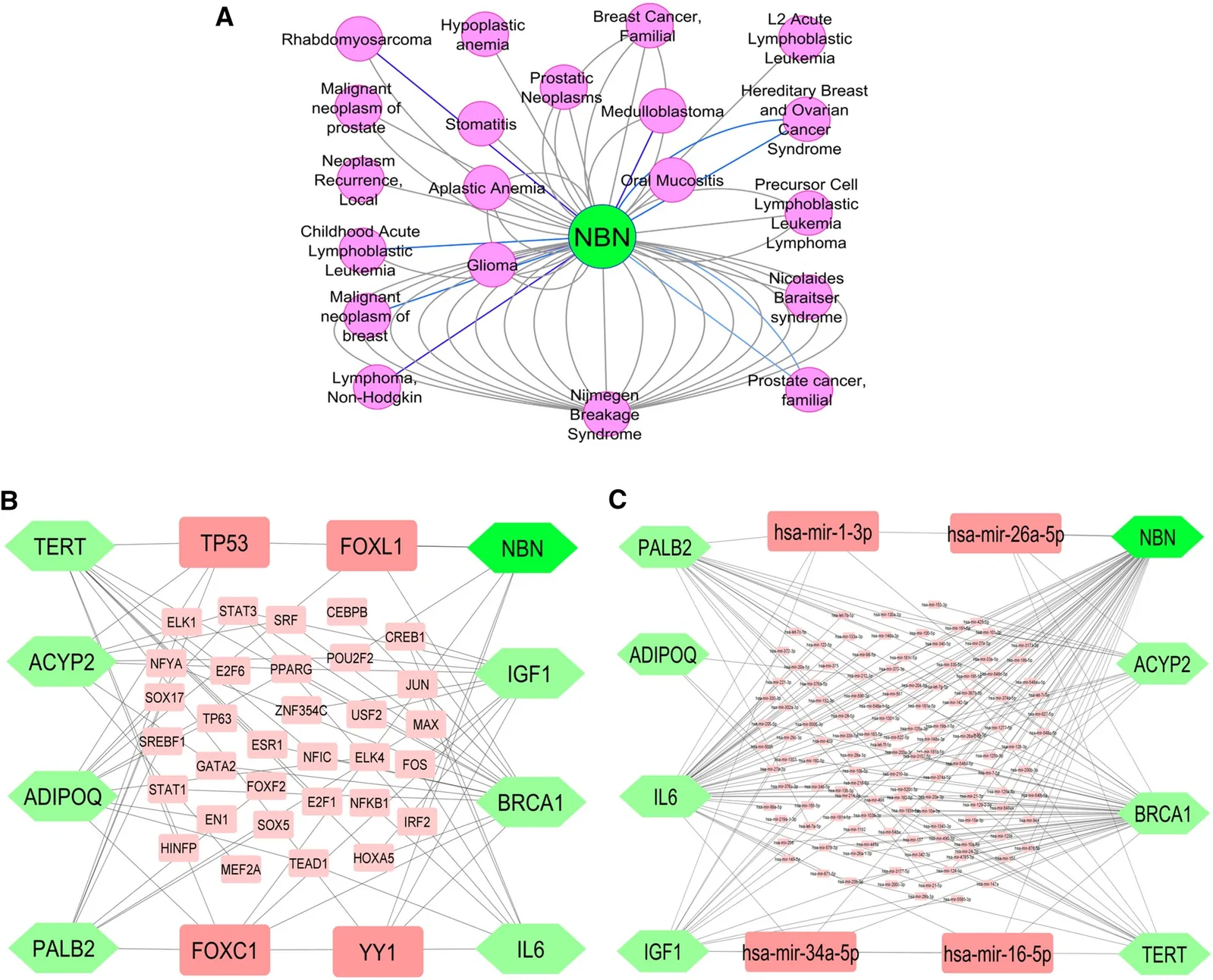
Comprehensive interaction networks highlighting the role of the NBN gene in cancer. (A) Disease-gene interaction network, mapping the association of NBN with various cancers, demonstrating its broad impact across cancer types. The green circular node represents the NBN gene, while pink circular nodes denote associated diseases or cancer phenotypes. (B) Transcription factor network, identifying TFs like TP53 and FOXL1 that regulate NBN and other cancer-related genes, underscoring their central role in gene regulation, where green diamond-shaped nodes represent cancer-associated genes (NBN, TERT, ACYP2, ADIPOQ, IGF1, BRCA1, PALB2, IL6), and pink rectangular nodes represent transcription factors (TFs) regulating these genes. (C) miRNA interaction network, showing miRNAs such as hsa-mir-192-5p that target NBN and other genes, highlighting their importance in post-transcriptional regulation. Green diamond-shaped nodes indicate target genes, and pink rectangular nodes represent miRNAs involved in post-transcriptional regulation. Edges indicate regulatory or interaction relationships. Collectively, these networks provide a systems-level view of NBN-centered interactions across disease associations, transcriptional control, and post-transcriptional regulation in oncogenesis.

To support the findings of statistical meta-analysis results and provide additional biological context regarding the potential role of NBN in multiple cancers, we performed an exploratory bioinformatics analysis using multiple online public databases and computational tools. In order to investigate the association types (direct/indirect) of NBN gene with breast, head and neck, bladder and brain cancers detected by meta-analysis, expression analysis was performed. Box-plots analysis (Fig. 5) showed significant mean/median expression differences between some cancers and normal tissues, suggesting that NBN gene might be directly associated with different cancers (BRCA, COAD, DLBC, HNSC, LAML, LIHC, LGG, SKCM), though expression patterns varied across cancer types. These results indicate that NBN gene may be directly/indirectly associated with the breast cancer (BRCA) and head and neck cancer (HNSC), but indirectly associated with the bladder and brain cancers. Thus, expression analysis of NBN gene further clarify the types of association with different cancers detected by the meta-analysis. The disease-gene network analysis (Fig. 6A and Table S5) indicated that the NBN gene is significantly associated with various cancer-related disease including bladder, nasopharyngeal, brain, BRCA1, ADIPOQ, IGF1, ACYP2, TERT, and PALB2 that also supported the meta-analysis results. The gene regulatory network analysis identified 4 transcription factors (TP53, FOXL1, FOXC1, and YY1) as the transcriptional regulators that are involved in DNA damage repair, cell cycle regulation, and tumor suppression (Fig. 6B) [99–102], and four microRNAs (hsa-mir-192-5p, hsa-mir-215-5p, and hsa-mir-132-3p) as the post-transcriptional regulators that might regulate NBN gene for the development and progression of different cancers (Fig. 6C) [103–105]. The functional enrichment analyses with GO-terms and KEGG-pathways revealed some crucial mechanisms of NBN gene that are associated with the development and progression of multiple cancers (Table S6). This analysis confirmed that NBN is significantly involved in vital DNA repair pathways such as homologous recombination, non-homologous end joining (NHEJ), DNA replication, and telomere maintenance, while also interacting with tumor suppressor p53 signaling [106–121]. The enrichment of molecular functions, including protein binding, damaged DNA binding, and protein N-terminus binding, alongside its localization in critical cellular components such as the PML body, telomeric region, nucleoplasm, and Mre11 complex, emphasizes its essential role in genomic stability. KEGG pathway analysis further highlighted homologous recombination and cellular senescence among the pathways associated with NBN, suggesting that NBN-related networks may be relevant to cancer biology. Collectively, these exploratory findings provide biological plausibility for the genetic associations observed in the meta-analysis, while emphasizing that experimental and clinical validation is required to determine causal mechanisms or potential biomarker/therapeutic relevance.

## Discussion and conclusion

At first, we tried to make a consensus decision about the association of NBN gene polymorphisms (rs1805794, rs709816) with multiple cancer risks by placing the emphasis on a larger sample size and appropriate statistical modeling and comparing with the previous meta-analysis results. To do it, we collected all available datasets through an online database search and previous meta-analyses, resulting in a larger sample size than existing studies [3–8]. We performed a statistical meta-analysis based on 58 single GWAS findings, which included 23 494 and 5325 cases and 29 592 and 11 149 control samples for rs1805794 (8360 G > C) and rs709816 (1197 T > C) polymorphisms, respectively. This meta-analysis suggested that the C allele of rs1805794 polymorphism is not statistically significantly associated with overall cancer risk under all the genetic models, which is consistent with previous meta-analysis reports [3, 4]. However, the C allele of NBN gene rs709816 polymorphism shows a significant association with overall cancer risk under only one genetic model (CC vs. TT). The observed association for rs709816 under the CC vs. TT model should be interpreted with caution, as it is marginally significant, uncorrected for multiple testing, indicating that the finding is exploratory rather than conclusive. Subgroup analysis based on ethnicity indicates that the C allele of both rs1805794 and rs709816 polymorphisms is not significantly associated with overall cancer for all populations and genetic models, except the mixed population for rs709816 under the allelic model only. These findings are partially supported by previous meta-analyses [3, 4]. In the cancer-specific subgroup analysis, the C allele of rs1805794 is significantly associated with increasing the risk of bladder cancer under three genetic models, which is aligned by the previous report [5]. Additionally, the C allele of rs1805794 is significantly associated with nasopharyngeal cancer risk under four genetic models, findings are supported by a previous study [3], and brain cancer risk for only allelic model (C vs. G). Moreover, the C allele of rs709816 polymorphism shows a significant association with breast cancer risk under the homozygote model (CC vs. TT), while it is insignificantly associated with bone cancer, both association statuses are consistent with the previous meta-analysis report [6]. Another study reported that NBN mRNA expression was significantly upregulated in peripheral blood cells (PBCs) of patients with hereditary breast and ovarian cancer (HBOC) compared with healthy controls [122], and that NBN expression was positively correlated with BRCA1, PALB2, and BARD1. Notably, increased BRCA1 expression was also observed in BRCA-associated breast cancer tissues compared with normal breast cells [122, 123], suggesting that NBN may act as a co-regulatory gene in breast cancer. Subgroup analysis based on the source of the control population shows a significant association with overall cancer for the hospital-based control populations. Though previous studies reported significant associations between rs1805794 and lung or blood cancer [4, 8], our updated analysis did not confirm these associations. This meta-analysis demonstrated the inclusion of additional datasets and improved statistical power that reduced the influence of study-specific effects. This suggests that earlier positive findings may have been driven by limited sample sizes or population-specific factors. Therefore, our results provide a more refined and updated consensus on the role of NBN polymorphisms in cancers, emphasizing cancer-type–specific effects rather than overall associations.

Publication bias analysis is used to evaluate whether the published studies included in this meta-analysis are systematically skewed toward significant results, which could distort the true effect estimate. Although funnel plot symmetry and Begg’s test suggested no publication bias for either polymorphism, Egger’s test indicated potential bias for rs709816 under the allelic (C vs. T) and homozygote (CC vs. TT) models. This discrepancy may be attributable to the limited number of included studies and the higher sensitivity of Egger’s test to small study effects. Therefore, the association results for rs709816 under these models should be interpreted with caution. Therefore, this study incorporated a larger dataset and a refined statistical approach, including FPRP analysis and meta-regression, enabling the investigation of statistical power and a systematic assessment of potential confounders and subgroup-specific effects. While previous meta-analyses [3–8] have reported mixed findings, this study’s results emphasize the importance of considering heterogeneity sources when evaluating genetic associations. By accounting for population-specific effects and study design factors, this study offers a more comprehensive perspective on the role of NBN gene polymorphisms in cancer susceptibility.

To complement the statistical meta-analysis and gain deeper biological insights, we conducted a bioinformatics analysis that provided supportive evidence for an association of NBN with multiple cancer types, including bladder, nasopharyngeal, brain, prostate, and breast cancers. Differentially expressed genes (DEGs) identified between cancer and control groups are often directly/indirectly associated with cancer development or progression [124, 125]. Some non-DEGs between cancer and control groups are indirectly associated with the development or progression of cancer [126, 127]. Interaction network analysis identified key transcriptional regulators (TP53, FOXL1, FOXC1, and YY1) [99–102] and microRNAs (hsa-miR-192-5p, hsa-miR-215-5p, and hsa-miR-132-3p) [103–105]. These TFs and miRNAs are notable because they act as key regulatory hubs by potentially regulating the expression of the NBN gene through transcriptional and post-transcriptional mechanisms, thereby influencing critical cancer-related processes such as DNA damage repair, cell cycle regulation, apoptosis, and tumor progression. Functional enrichment analyses further indicated that NBN is enriched in DNA repair–related pathways, including homologous recombination and non-homologous end joining (NHEJ), as well as cellular senescence, which is consistent with its established role in maintaining genomic stability. However, these bioinformatics findings are exploratory and do not establish causality or clinical relevance.

These findings may contribute to a better understanding of the potential role of NBN polymorphisms in cancer susceptibility and could inform future hypothesis-driven research on genetic risk stratification [122, 123]. In addition, the identification of regulatory factors associated with NBN highlights candidate pathways for further functional investigation, particularly in cancers characterized by impaired DNA repair mechanisms [128]. Future studies integrating NBN genetic variation with experimental and clinical data will be necessary to clarify their biological relevance and to determine whether such associations have any translational significance in precision oncology. While previous studies have demonstrated that genetic variants and differentially expressed genes can serve as diagnostic [129–133] or prognostic [134–136] biomarkers in cancer [133, 136, 137], the findings of the present study should be regarded as associative and exploratory, highlighting potential biological relevance rather than established clinical utility.

Although rs1805794 and rs709816 are not classified as highly deleterious variants, they are located in functionally relevant regions of the NBN gene and may influence gene regulation or protein activity involved in DNA damage repair. Current evidence suggests that these polymorphisms exert modest biological effects, potentially modifying cancer susceptibility through subtle regulatory mechanisms rather than direct loss of function. However, functional validation studies remain limited, and further experimental investigations are needed to clarify their precise molecular roles. This study provides an updated and comprehensive evaluation of NBN polymorphisms across multiple cancer types, the findings should be interpreted with caution. While the meta-analysis has several strengths, certain limitations may impact the final findings. The primary limitations include: (i) a limited number of datasets for certain cancer types, which may reduce the statistical power of the associations, (ii) the absence of some cancer types, potentially influencing the overall assessment of gene polymorphisms and overall cancer risk, (iii) unaccounted heterogeneity factors, such as age, sex, smoking, alcohol consumption, dietary habits, and environmental exposures, which may introduce variability and impact the associations observed, (iv) given the large number of genetic models, subgroup analyses, and cancer types evaluated, multiple statistical comparisons were performed, increasing the potential for false-positive results, and (v) some subgroup associations observed for specific cancer types, may be influenced by multiple testing, and therefore should be interpreted cautiously as exploratory rather than definitive findings. Additionally, (i) the further limitation is that gene–gene and gene–environment interactions were not examined, and therefore the complex multifactorial nature of cancer susceptibility may not be fully captured by single-gene association analyses, and (ii) the bioinformatics analyses, including differential expression and regulatory network predictions, indicate potential associations but do not establish causal or mechanistic relationships, as the identified transcription factor and miRNA interactions are computationally inferred and require experimental validation.

In conclusion, this study integrates meta-analysis and bioinformatics to assess the association between NBN gene polymorphisms and cancer risk. The statistical meta-analysis confirmed significant associations, particularly with bladder, nasopharyngeal, brain, and breast cancers, while subgroup analyses highlighted population-specific effects. Bioinformatics analysis further validated these findings, showing NBN’s enrichment in DNA repair pathways and its regulation by key transcription factors (TP53, FOXL1, FOXC1, YY1) and microRNAs (hsa-miR-192-5p, hsa-miR-215-5p, hsa-miR-132-3p). However, these findings should be interpreted cautiously, as several associations are modest and exploratory, and the bioinformatics results are based on computational inference rather than experimental validation. Overall, this work offers an updated synthesis of existing evidence on NBN polymorphisms in cancer risk, while underscoring the need for further large-scale and functional studies to clarify their biological and clinical relevance.

## Supplementary Material

bpag012_Supplementary_Data

## Data Availability

All data generated or analyzed during this study are included in this published article. The R scripts used for data processing and meta-analysis (including the meta package) are available from the corresponding author upon reasonable request to facilitate reproducibility and transparency.
