# Supplementary material for: Investigating the association of NBN gene polymorphisms with multiple cancers through statistical meta-analysis and bioinformatics insights
Source: Biol Methods Protoc. 2026 Feb 27;11(1):bpag012. doi: 10.1093/biomethods/bpag012 (PMC12967219; doi:10.1093/biomethods/bpag012)
Supplement: bpag012_Supplementary_Data [file bpag012_supplementary_data.zip › Supplementary_file.pdf]

Supplementary Information

Table S1. Heterogeneity analysis of the NBN gene polymorphisms (rs1805794, rs709816).

Types of Diseases	Genetic Model	Study number	Model	OR [95% CI]	Z-val	p-val	Q	d.f	p-val (Q)	Tau2	I2 %
rs1805794											
Overall	C vs. G	67	REM	1.06 [1; 1.12]	1.85	0.0645	328.03	66	0.000	0.04	79.88
	CC vs. GG	67	REM	1.13 [0.99; 1.29]	1.76	0.0784	316.04	66	0.000	0.21	79.12
	CG vs. GG	67	REM	1.05 [0.98; 1.11]	1.39	0.1631	139.33	66	0.000	0.03	52.63
	CC + CG vs. GG	67	REM	1.07 [0.99; 1.15]	1.72	0.0862	218.77	66	0.000	0.06	69.83
	CC vs. CG + GG	67	REM	1.1 [0.98; 1.23]	1.65	0.0993	265.04	66	0.000	0.13	75.1
Breast Cancer	C vs. G	16	FEM	1 [0.95; 1.05]	0.13	0.8995	16.25	15	0.366	0	7.68
	CC vs. GG	16	FEM	0.99 [0.89; 1.11]	-0.11	0.9126	18.06	15	0.260	0.01	16.94
	CG vs. GG	16	FEM	1.01 [0.94; 1.09]	0.39	0.6942	12.27	15	0.658	0	0
	CC + CG vs. GG	16	FEM	1.01 [0.94; 1.08]	0.28	0.7773	13.43	15	0.569	0	0
	CC vs. CG + GG	16	FEM	0.99 [0.9; 1.1]	-0.16	0.8712	18.88	15	0.219	0.01	20.55
Bladder Cancer	C vs. G	6	FEM	1.09 [1.01; 1.16]	2.34	0.0193	3.27	5	0.659	0	0
	CC vs. GG	6	FEM	1.15 [0.98; 1.33]	1.75	0.0794	6.32	5	0.277	0	20.85
	CG vs. GG	6	FEM	1.13 [1.02; 1.24]	2.35	0.0186	1.9	5	0.863	0	0
	CC + CG vs. GG	6	FEM	1.13 [1.03; 1.24]	2.54	0.0110	1.49	5	0.914	0	0
	CC vs. CG + GG	6	FEM	1.07 [0.93; 1.24]	0.99	0.3216	8.05	5	0.153	0	37.92
Ovarian Cancer	C vs. G	4	FEM	0.95 [0.86; 1.04]	-1.16	0.2454	0.84	3	0.840	0	0
	CC vs. GG	4	FEM	0.85 [0.69; 1.05]	-1.5	0.1326	0.47	3	0.925	0	0
	CG vs. GG	4	FEM	1 [0.87; 1.14]	-0.04	0.9645	1.07	3	0.784	0	0
	CC + CG vs. GG	4	FEM	0.97 [0.85; 1.1]	-0.55	0.5842	1.07	3	0.784	0	0
	CC vs. CG + GG	4	FEM	0.85 [0.69; 1.04]	-1.59	0.1123	0.3	3	0.959	0	0
Skin Cancer	C vs. G	2	FEM	1.1 [0.96; 1.26]	1.32	0.1852	0.8	1	0.372	0	0
	CC vs. GG	2	FEM	1.2 [0.9; 1.61]	1.22	0.2209	0.58	1	0.447	0	0

	CG vs. GG	2	FEM	1.09 [0.89; 1.33]	0.81	0.4169	0.42	1	0.519	0	0
	CC + CG vs. GG	2	FEM	1.11 [0.92; 1.35]	1.11	0.2653	0.64	1	0.425	0	0
	CC vs. CG + GG	2	FEM	1.15 [0.88; 1.52]	1.01	0.3106	0.34	1	0.560	0	0
Lung Cancer	C vs. G	6	FEM	1.01 [0.9; 1.13]	0.18	0.8595	8.57	5	0.128	0.01	41.62
	CC vs. GG	6	REM	0.88 [0.55; 1.41]	-0.53	0.5945	11.56	5	0.041	0.19	56.74
	CG vs. GG	6	FEM	1.05 [0.88; 1.25]	0.56	0.5748	6.72	5	0.243	0	25.56
	CC + CG vs. GG	6	FEM	1.05 [0.89; 1.24]	0.59	0.5560	7.58	5	0.181	0	34.03
	CC vs. CG + GG	6	REM	0.96 [0.67; 1.38]	-0.23	0.8192	10.02	5	0.075	0.1	50.1
Blood Cancer	C vs. G	13	REM	1.1 [0.9; 1.35]	0.94	0.3458	98.34	12	0.000	0.11	87.8
	CC vs. GG	13	REM	1.47 [0.93; 2.33]	1.65	0.0981	68.21	12	0.000	0.45	82.41
	CG vs. GG	13	REM	1.03 [0.81; 1.3]	0.22	0.8288	46.52	12	0.000	0.12	74.21
	CC + CG vs. GG	13	REM	1.11 [0.84; 1.47]	0.76	0.4478	71.77	12	0.000	0.19	83.28
	CC vs. CG + GG	13	REM	1.34 [0.95; 1.88]	1.65	0.0992	50.05	12	0.000	0.25	76.02
Prostate Cancer	C vs. G	2	FEM	1.09 [0.88; 1.36]	0.81	0.4201	1.06	1	0.304	0	5.41
	CC vs. GG	2	FEM	0.98 [0.62; 1.55]	-0.08	0.9351	0.84	1	0.358	0	0
	CG vs. GG	2	FEM	1.48 [1.07; 2.04]	2.37	0.0179	0.91	1	0.341	0	0
	CC + CG vs. GG	2	FEM	1.33 [0.98; 1.8]	1.86	0.0634	1.18	1	0.276	0.01	15.59
	CC vs. CG + GG	2	FEM	0.8 [0.53; 1.22]	-1.03	0.3029	0.33	1	0.566	0	0
Colon Cancer	C vs. G	4	FEM	1 [0.9; 1.11]	-0.01	0.9932	2.87	3	0.412	0	0
	CC vs. GG	4	FEM	0.95 [0.77; 1.18]	-0.46	0.6491	4.02	3	0.259	0.04	25.46
	CG vs. GG	4	FEM	1.09 [0.94; 1.27]	1.12	0.2646	0.87	3	0.833	0	0
	CC + CG vs. GG	4	FEM	1.05 [0.91; 1.22]	0.68	0.4994	1.71	3	0.635	0	0
	CC vs. CG + GG	4	FEM	0.91 [0.75; 1.11]	-0.91	0.3615	3.58	3	0.311	0.03	16.09
Laryngeal Cancer	C vs. G	2	FEM	1.01 [0.85; 1.2]	0.08	0.9332	0.25	1	0.620	0	0
	CC vs. GG	2	FEM	1.12 [0.76; 1.64]	0.57	0.5691	0.07	1	0.798	0	0
	CG vs. GG	2	FEM	0.9 [0.69; 1.18]	-0.76	0.4495	2.56	1	0.110	0.06	60.9
	CC + CG vs. GG	2	FEM	0.94 [0.73; 1.22]	-0.44	0.6572	1.5	1	0.221	0.02	33.37

	CC vs. CG + GG	2	FEM	1.15 [0.81; 1.62]	0.78	0.4379	0.75	1	0.386	0	0
Others	C vs. G	4	REM	0.97 [0.7; 1.33]	-0.19	0.8463	27.71	3	0.000	0.09	89.17
	CC vs. GG	4	REM	1.03 [0.46; 2.29]	0.07	0.9417	32.66	3	0.000	0.55	90.81
	CG vs. GG	4	REM	0.85 [0.62; 1.16]	-1.04	0.3000	9.17	3	0.027	0.06	67.3
	CC + CG vs. GG	4	REM	0.88 [0.59; 1.31]	-0.64	0.5198	19.41	3	0.000	0.13	84.55
	CC vs. CG + GG	4	REM	1.13 [0.59; 2.19]	0.37	0.7121	23.59	3	0.000	0.36	87.28
Brain Cancer	C vs. G	2	REM	1.2 [0.79; 1.82]	0.86	0.3885	11.5	1	0.001	0.08	91.3
	CC vs. GG	2	REM	1.5 [0.91; 2.47]	1.61	0.1079	3.8	1	0.051	0.1	73.65
	CG vs. GG	2	REM	1.06 [0.62; 1.81]	0.22	0.8247	8.28	1	0.004	0.13	87.93
	CC + CG vs. GG	2	REM	1.17 [0.65; 2.11]	0.53	0.5985	11.92	1	0.001	0.17	91.61
	CC vs. CG + GG	2	FEM	1.49 [1.2; 1.84]	3.66	0.0003	0.93	1	0.336	0	0
Nasopharyngeal Cancer	C vs. G	4	REM	1.56 [1.1; 2.2]	2.51	0.0120	13.21	3	0.004	0.09	77.3
	CC vs. GG	4	REM	2.28 [1.06; 4.89]	2.12	0.0342	14.28	3	0.003	0.46	78.99
	CG vs. GG	4	FEM	1.58 [1.32; 1.9]	4.9	0.0000	2.08	3	0.557	0	0
	CC + CG vs. GG	4	FEM	1.98 [1.66; 2.35]	7.76	0.0000	5.92	3	0.116	0.04	49.33
	CC vs. CG + GG	4	REM	1.85 [0.93; 3.69]	1.75	0.0800	13.9	3	0.003	0.37	78.42
Bone Cancer	C vs. G	2	FEM	1.06 [0.84; 1.33]	0.48	0.6346	1.42	1	0.233	0.01	29.73
	CC vs. GG	2	FEM	2.65 [0.16; 43.47]	0.68	0.4941	0	1	0.949	0	0
	CG vs. GG	2	REM	0.96 [0.43; 2.14]	-0.1	0.9225	3.75	1	0.053	0.25	73.32
	CC + CG vs. GG	2	REM	0.96 [0.43; 2.14]	-0.1	0.9225	3.75	1	0.053	0.25	73.32
	CC vs. CG + GG	2	FEM	2.94 [0.18; 47.86]	0.76	0.4488	0.09	1	0.764	0	0
Asian	C vs. G	15	REM	1.22 [0.99; 1.49]	1.89	0.0589	241.81	14	0.000	0.15	94.21
	CC vs. GG	15	REM	1.53 [0.97; 2.41]	1.82	0.0683	230.45	14	0.000	0.67	93.92
	CG vs. GG	15	REM	1.2 [1; 1.45]	1.92	0.0550	68.35	14	0.000	0.1	79.52
	CC + CG vs. GG	15	REM	1.29 [0.99; 1.68]	1.92	0.0551	142.88	14	0.000	0.22	90.2
	CC vs. CG + GG	15	REM	1.34 [0.97; 1.84]	1.8	0.0726	175.79	14	0.000	0.33	92.04
Caucasian	C vs. G	43	FEM	1.02 [0.98; 1.05]	1.03	0.3047	40.29	42	0.546	0	0

	CC vs. GG	43	FEM	1.05 [0.97; 1.14]	1.23	0.2169	53.29	42	0.114	0.02	21.19
	CG vs. GG	43	FEM	1 [0.95; 1.05]	0.12	0.9076	39.06	42	0.601	0	0
	CC + CG vs. GG	43	FEM	1.01 [0.97; 1.06]	0.53	0.5953	35.32	42	0.757	0	0
	CC vs. CG + GG	43	REM	1.05 [0.95; 1.16]	1.02	0.3079	63.73	42	0.017	0.03	34.1
Mixed	C vs. G	9	FEM	0.98 [0.91; 1.06]	-0.46	0.6483	10.03	8	0.263	0	20.26
	CC vs. GG	9	FEM	0.93 [0.77; 1.12]	-0.79	0.4292	1.22	8	0.996	0	0
	CG vs. GG	9	REM	1.1 [0.9; 1.35]	0.91	0.3654	21.96	8	0.005	0.06	63.57
	CC + CG vs. GG	9	REM	1.06 [0.88; 1.26]	0.6	0.5513	18.91	8	0.015	0.04	57.69
	CC vs. CG + GG	9	FEM	0.9 [0.77; 1.06]	-1.27	0.2051	1.3	8	0.996	0	0
PB	C vs. G	33	REM	1.01 [0.95; 1.09]	0.37	0.713	91.91	32	0	0.02	65.18
	CC vs. GG	33	REM	1.02 [0.87; 1.19]	0.23	0.8149	82.76	32	0	0.11	61.33
	CG vs. GG	33	REM	1.02 [0.94; 1.1]	0.46	0.6443	57.57	32	0.0037	0.02	44.42
	CC + CG vs. GG	33	REM	1.02 [0.93; 1.12]	0.42	0.6755	75.39	32	0	0.04	57.55
	CC vs. CG + GG	33	REM	1.02 [0.9; 1.15]	0.24	0.811	63.82	32	0.0007	0.05	49.86
HB	C vs. G	32	REM	1.11 [1; 1.23]	1.99	0.0461	225.41	31	0	0.06	86.25
	CC vs. GG	32	REM	1.27 [1.01; 1.6]	2.04	0.041	220.09	31	0	0.31	85.92
	CG vs. GG	32	REM	1.07 [0.97; 1.19]	1.34	0.1787	80.51	31	0	0.05	61.5
	CC + CG vs. GG	32	REM	1.12 [0.99; 1.28]	1.83	0.0679	138.98	31	0	0.09	77.69
	CC vs. CG + GG	32	REM	1.2 [0.99; 1.45]	1.82	0.0695	188.82	31	0	0.22	83.58
Mixed	C vs. G	2	FEM	1.04 [0.94; 1.15]	0.72	0.4725	0.48	1	0.4885	0	0
	CC vs. GG	2	FEM	1.04 [0.83; 1.29]	0.33	0.7409	1.08	1	0.2981	0	7.63
	CG vs. GG	2	FEM	1.11 [0.95; 1.29]	1.29	0.1955	0.06	1	0.8132	0	0
	CC + CG vs. GG	2	FEM	1.09 [0.94; 1.26]	1.13	0.2576	0.26	1	0.6102	0	0
	CC vs. CG + GG	2	FEM	0.98 [0.81; 1.2]	-0.15	0.8805	0.91	1	0.3388	0	0
rs709816											
Overall	C vs. G	15	FEM	0.95 [0.90; 1.00]	-1.99	0.0469	9.28	14	0.8126	0	0
	CC vs. GG	15	FEM	0.91 [0.82; 1.01]	-1.81	0.0709	11.91	14	0.6133	0	0

	CG vs. GG	15	FEM	0.97 [0.88; 1.07]	-0.62	0.5335	14.75	14	0.3957	0	5.06
	CC + CG vs. GG	15	FEM	0.94 [0.86; 1.04]	-1.22	0.221	12.06	14	0.6015	0	0
	CC vs. CG + GG	15	FEM	0.93 [0.87; 1]	-1.91	0.0556	11.24	14	0.6672	0	0
Others	C vs. G	6	FEM	0.96 [0.91; 1.03]	-1.12	0.2623	3.66	5	0.5992	0	0
	CC vs. GG	6	FEM	0.95 [0.83; 1.07]	-0.86	0.3875	2.39	5	0.7931	0	0
	CG vs. GG	6	FEM	1.03 [0.91; 1.16]	0.44	0.6615	4.04	5	0.5443	0	0
	CC + CG vs. GG	6	FEM	0.99 [0.88; 1.11]	-0.15	0.8799	2.16	5	0.8272	0	0
	CC vs. CG + GG	6	FEM	0.93 [0.85; 1.02]	-1.5	0.1331	7.57	5	0.1815	0	33.96
Blood Cancer	C vs. G	4	FEM	0.96 [0.85; 1.09]	-0.58	0.561	2.47	3	0.4814	0	0
	CC vs. GG	4	FEM	0.93 [0.7; 1.22]	-0.54	0.59	1.84	3	0.607	0	0
	CG vs. GG	4	FEM	0.96 [0.74; 1.26]	-0.26	0.7916	1.18	3	0.7566	0	0
	CC + CG vs. GG	4	FEM	0.95 [0.73; 1.22]	-0.42	0.674	1.21	3	0.7505	0	0
	CC vs. CG + GG	4	FEM	0.95 [0.8; 1.14]	-0.52	0.6056	3.06	3	0.3819	0	2.07
Breast Cancer	C vs. G	3	FEM	0.88 [0.77; 0.99]	-2.1	0.0362	1.06	2	0.5894	0	0
	CC vs. GG	3	REM	0.65 [0.33; 1.27]	-1.27	0.2054	5.24	2	0.0728	0.24	61.83
	CG vs. GG	3	REM	0.55 [0.24; 1.26]	-1.41	0.1588	4.83	2	0.0892	0.32	58.62
	CC + CG vs. GG	3	REM	0.65 [0.34; 1.26]	-1.27	0.2032	5.27	2	0.0719	0.23	62.02
	CC vs. CG + GG	3	FEM	0.9 [0.75; 1.07]	-1.2	0.2309	0.2	2	0.9063	0	0
Bone Cancer	C vs. G	2	FEM	0.99 [0.79; 1.25]	-0.08	0.9358	0.06	1	0.8098	0	0
	CC vs. GG	2	FEM	1.01 [0.61; 1.67]	0.05	0.9618	0.14	1	0.7083	0	0
	CG vs. GG	2	FEM	1.39 [0.09; 22.55]	0.23	0.8178	0.62	1	0.4325	0	0
	CC + CG vs. GG	2	FEM	1.01 [0.61; 1.67]	0.05	0.9604	0.13	1	0.7206	0	0
	CC vs. CG + GG	2	FEM	0.97 [0.71; 1.33]	-0.17	0.8656	0.07	1	0.7978	0	0
Caucasian	C vs. G	7	FEM	0.97 [0.91; 1.04]	-0.85	0.3954	5	6	0.5437	0	0
	CC vs. GG	7	FEM	0.95 [0.82; 1.09]	-0.76	0.4447	8.15	6	0.2276	0	26.35
	CG vs. GG	7	FEM	0.97 [0.84; 1.12]	-0.44	0.6618	8.01	6	0.2372	0	25.12
	CC + CG vs. GG	7	FEM	0.96 [0.84; 1.1]	-0.59	0.5522	7.54	6	0.2739	0	20.41

	CC vs. CG + GG	7	FEM	0.97 [0.88; 1.06]	-0.75	0.4544	3.68	6	0.7197	0	0
Asian	C vs. G	3	FEM	0.92 [0.81; 1.05]	-1.27	0.2051	0.98	2	0.6117	0	0
	CC vs. GG	3	FEM	0.82 [0.62; 1.09]	-1.35	0.1771	1.34	2	0.5121	0.02	0
	CG vs. GG	3	FEM	0.86 [0.67; 1.1]	-1.22	0.2237	1.35	2	0.51	0.02	0
	CC + CG vs. GG	3	FEM	0.84 [0.66; 1.07]	-1.41	0.1581	1.48	2	0.4764	0.04	0
	CC vs. CG + GG	3	FEM	0.92 [0.76; 1.12]	-0.82	0.4109	0.21	2	0.9025	0	0
Mixed	C vs. G	5	FEM	0.93 [0.85; 1.02]	-1.58	0.1142	2.42	4	0.6589	0	0
	CC vs. GG	5	FEM	0.88 [0.74; 1.06]	-1.32	0.1865	1.39	4	0.8466	0	0
	CG vs. GG	5	FEM	1.02 [0.87; 1.21]	0.27	0.7855	4.03	4	0.4021	0.01	0.72
	CC + CG vs. GG	5	FEM	0.97 [0.83; 1.13]	-0.44	0.6629	1.92	4	0.7502	0	0
	CC vs. CG + GG	5	FEM	0.87 [0.75; 1]	-2.01	0.0444	5.72	4	0.2214	0	30.03
PB	C vs. G	3	FEM	0.97 [0.85; 1.1]	-0.54	0.5878	0.3	2	0.8586	0	0
	CC vs. GG	3	FEM	0.92 [0.71; 1.2]	-0.61	0.5451	0.29	2	0.8634	0	0
	CG vs. GG	3	FEM	0.91 [0.69; 1.2]	-0.65	0.5168	0.36	2	0.8348	0	0
	CC + CG vs. GG	3	FEM	0.92 [0.72; 1.18]	-0.66	0.5084	0.19	2	0.9075	0	0
	CC vs. CG + GG	3	FEM	0.98 [0.82; 1.16]	-0.28	0.7813	0.22	2	0.8978	0	0
HB	C vs. G	6	FEM	0.9 [0.81; 1]	-1.92	0.0544	4.02	5	0.5472	0	0
	CC vs. GG	6	FEM	0.79 [0.62; 1]	-1.98	0.0475	7.83	5	0.1661	0.13	36.1
	CG vs. GG	6	FEM	0.8 [0.63; 1.02]	-1.79	0.0736	7.43	5	0.1907	0.3	32.69
	CC + CG vs. GG	6	FEM	0.82 [0.67; 1]	-1.91	0.0556	7.53	5	0.1842	0	33.59
	CC vs. CG + GG	6	FEM	0.9 [0.77; 1.05]	-1.34	0.1808	3	5	0.6993	0	0
Mixed	C vs. G	5	FEM	0.93 [0.85; 1.02]	-1.58	0.1142	2.42	4	0.6589	0	0
	CC vs. GG	5	FEM	0.88 [0.74; 1.06]	-1.32	0.1865	1.39	4	0.8466	0	0
	CG vs. GG	5	FEM	1.02 [0.87; 1.21]	0.27	0.7855	4.03	4	0.4021	0.01	0.72
	CC + CG vs. GG	5	FEM	0.97 [0.83; 1.13]	-0.44	0.6629	1.92	4	0.7502	0	0
	CC vs. CG + GG	5	FEM	0.87 [0.75; 1]	-2.01	0.0444	5.72	4	0.2214	0	30.03

Table S2. Meta-regression analysis results for the main sources of heterogeneity in existing heterogeneous (I^2 > 50%) subgroups.

rs1805794: Overall cancer risks							
Model	Variable	Estimate	SE	Zval	Pval	CI_lb	CI_ub
C vs. G	Intercept	16.30	23.26	0.70	0.483	-29.29	61.89
	Year	-0.01	0.01	-0.68	0.497	-0.03	0.01
	Case_control_ratio C vs. G	0.11	0.18	0.61	0.540	-0.24	0.46
	Cancer Type (Blood Cancer)	0.07	0.19	0.38	0.705	-0.30	0.44
	Cancer Type (Bone Cancer)	-0.44	1.45	-0.30	0.764	-3.28	2.41
	Cancer Type (Brain Cancer)	-0.17	0.24	-0.69	0.489	-0.64	0.30
	Cancer Type (Breast Cancer)	-0.19	0.15	-1.26	0.208	-0.49	0.11
	Cancer Type (Colon Cancer)	-0.23	0.19	-1.22	0.222	-0.60	0.14
	Cancer Type (Head and neck Cancer)	0.01	0.49	0.01	0.992	-0.96	0.97
	Cancer Type (Kidney Cancer)	-0.13	0.39	-0.34	0.737	-0.91	0.64
	Cancer Type (Laryngeal Cancer)	-0.36	0.27	-1.36	0.173	-0.88	0.16
	**Cancer Type (Liver Cancer)**	**-1.09**	**0.21**	**-5.10**	**0.000**	**-1.52**	**-0.67**
	Cancer Type (Lung Cancer)	-0.30	0.21	-1.43	0.151	-0.70	0.11
	Cancer Type (Nasopharyngeal Cancer)	0.29	0.20	1.39	0.163	-0.12	0.69
	Cancer Type (Ovarian Cancer)	-0.13	0.20	-0.64	0.523	-0.53	0.27
	Cancer Type (Prostate Cancer)	0.05	0.39	0.13	0.897	-0.72	0.82
	Cancer Type (Skin Cancer)	-0.03	0.22	-0.13	0.893	-0.45	0.40
	Cancer Type (Thyroid Cancer)	-0.07	0.48	-0.14	0.888	-1.02	0.88
	**Ethnicity (Caucasian)**	**-0.36**	**0.10**	**-3.54**	**0.000**	**-0.56**	**-0.16**
	**Ethnicity (Mixed)**	**-0.37**	**0.15**	**-2.39**	**0.017**	**-0.67**	**-0.07**
	HWE (Yes)	-0.13	0.21	-0.65	0.517	-0.54	0.27
	Source of Control (Mixed)	-0.19	0.22	-0.84	0.399	-0.63	0.25
	Source of Control (PB)	-0.02	0.11	-0.21	0.832	-0.24	0.20
CC vs. GG	Intercept	15.41	30.00	0.51	0.607	-43.39	74.21
	Year	-0.01	0.01	-0.49	0.626	-0.04	0.02
	Case_control_ratio CC vs. GG	0.28	0.23	1.21	0.225	-0.17	0.73
	Cancer Type (Blood Cancer)	0.31	0.25	1.23	0.220	-0.19	0.81
	Cancer Type (Bone Cancer)	0.33	1.49	0.22	0.822	-2.58	3.24
	Cancer Type (Brain Cancer)	-0.30	0.35	-0.87	0.383	-0.99	0.38
	Cancer Type (Breast Cancer)	-0.25	0.22	-1.13	0.256	-0.68	0.18
	Cancer Type (Colon Cancer)	-0.48	0.28	-1.73	0.084	-1.02	0.06
	Cancer Type (Head and neck Cancer)	0.39	0.54	0.73	0.468	-0.67	1.46
	Cancer Type (Kidney Cancer)	0.18	0.51	0.35	0.729	-0.83	1.19
	Cancer Type (Laryngeal Cancer)	-0.38	0.36	-1.07	0.285	-1.09	0.32
	**Cancer Type (Liver Cancer)**	**-1.55**	**0.38**	**-4.04**	**0.000**	**-2.30**	**-0.80**
	Cancer Type (Lung Cancer)	-0.38	0.28	-1.36	0.175	-0.92	0.17
	Cancer Type (Nasopharyngeal Cancer)	0.48	0.30	1.57	0.115	-0.12	1.07
	Cancer Type (Ovarian Cancer)	-0.20	0.29	-0.70	0.482	-0.77	0.36

	Cancer Type (Prostate Cancer)	-0.35	0.51	-0.68	0.494	-1.36	0.66
	Cancer Type (Skin Cancer)	-0.02	0.31	-0.07	0.946	-0.63	0.59
	Cancer Type (Thyroid Cancer)	-0.33	0.54	-0.61	0.542	-1.38	0.72
	**Ethnicity (Caucasian)**	**-0.47**	**0.14**	**-3.41**	**0.001**	**-0.75**	**-0.20**
	**Ethnicity (Mixed)**	**-0.55**	**0.22**	**-2.52**	**0.012**	**-0.98**	**-0.12**
	HWE (Yes)	-0.37	0.29	-1.29	0.195	-0.93	0.19
	Source of Control (Mixed)	-0.37	0.34	-1.09	0.277	-1.04	0.30
	Source of Control (PB)	-0.13	0.15	-0.91	0.363	-0.42	0.15
CG vs. GG	Intercept	13.48	18.85	0.71	0.475	-23.47	50.42
	Year	-0.01	0.01	-0.70	0.486	-0.02	0.01
	Case_control_ratio CG vs. GG	0.06	0.13	0.49	0.627	-0.19	0.32
	Cancer Type (Blood Cancer)	-0.16	0.15	-1.07	0.286	-0.45	0.13
	Cancer Type (Bone Cancer)	-0.19	0.27	-0.70	0.486	-0.71	0.34
	Cancer Type (Brain Cancer)	-0.19	0.18	-1.02	0.309	-0.55	0.17
	Cancer Type (Breast Cancer)	-0.19	0.12	-1.65	0.099	-0.43	0.04
	Cancer Type (Colon Cancer)	-0.14	0.15	-0.94	0.347	-0.45	0.16
	Cancer Type (Head and neck Cancer)	-0.14	0.35	-0.42	0.676	-0.82	0.53
	Cancer Type (Kidney Cancer)	-0.15	0.26	-0.57	0.570	-0.66	0.36
	Cancer Type (Laryngeal Cancer)	-0.37	0.21	-1.81	0.070	-0.78	0.03
	**Cancer Type (Liver Cancer)**	**-0.92**	**0.23**	**-3.96**	**0.000**	**-1.37**	**-0.46**
	Cancer Type (Lung Cancer)	-0.18	0.15	-1.16	0.245	-0.47	0.12
	Cancer Type (Nasopharyngeal Cancer)	0.13	0.18	0.75	0.453	-0.21	0.48
	Cancer Type (Ovarian Cancer)	-0.15	0.16	-0.96	0.335	-0.45	0.15
	Cancer Type (Prostate Cancer)	0.22	0.26	0.86	0.391	-0.29	0.73
	Cancer Type (Skin Cancer)	-0.05	0.18	-0.30	0.760	-0.40	0.29
	Cancer Type (Thyroid Cancer)	-0.03	0.30	-0.09	0.930	-0.61	0.56
	**Ethnicity (Caucasian)**	**-0.28**	**0.08**	**-3.40**	**0.001**	**-0.45**	**-0.12**
	**Ethnicity (Mixed)**	**-0.26**	**0.12**	**-2.18**	**0.029**	**-0.50**	**-0.03**
	HWE (Yes)	-0.01	0.11	-0.07	0.946	-0.23	0.21
	Source of Control (Mixed)	-0.14	0.18	-0.77	0.441	-0.50	0.22
	Source of Control (PB)	0.01	0.08	0.13	0.893	-0.15	0.18
CC + CG vs. GG	Intercept	16.90	19.91	0.85	0.396	-22.13	55.93
	Year	-0.01	0.01	-0.83	0.408	-0.03	0.01
	Case_control_ratio CC+CG vs. GG	0.11	0.15	0.77	0.443	-0.18	0.40
	Cancer Type (Blood Cancer)	-0.06	0.16	-0.37	0.713	-0.38	0.26
	Cancer Type (Bone Cancer)	-0.25	0.30	-0.85	0.396	-0.84	0.33
	Cancer Type (Brain Cancer)	-0.14	0.21	-0.67	0.501	-0.54	0.26
	Cancer Type (Breast Cancer)	-0.20	0.13	-1.52	0.129	-0.47	0.06
	Cancer Type (Colon Cancer)	-0.21	0.17	-1.21	0.224	-0.55	0.13
	Cancer Type (Head and neck Cancer)	-0.02	0.35	-0.05	0.963	-0.71	0.67
	Cancer Type (Kidney Cancer)	-0.05	0.27	-0.19	0.853	-0.59	0.49
	Cancer Type (Laryngeal Cancer)	-0.38	0.22	-1.69	0.090	-0.81	0.06

	**Cancer Type (Liver Cancer)**	**-1.10**	**0.24**	**-4.58**	**0.000**	**-1.57**	**-0.63**
	Cancer Type (Lung Cancer)	-0.21	0.17	-1.25	0.212	-0.53	0.12
	Cancer Type (Nasopharyngeal Cancer)	0.26	0.19	1.33	0.183	-0.12	0.63
	Cancer Type (Ovarian Cancer)	-0.17	0.17	-0.96	0.336	-0.51	0.17
	Cancer Type (Prostate Cancer)	0.13	0.28	0.48	0.634	-0.42	0.68
	Cancer Type (Skin Cancer)	-0.06	0.19	-0.29	0.771	-0.44	0.33
	Cancer Type (Thyroid Cancer)	-0.08	0.31	-0.28	0.783	-0.69	0.52
	**Ethnicity (Caucasian)**	**-0.35**	**0.09**	**-3.92**	**0.000**	**-0.52**	**-0.17**
	**Ethnicity (Mixed)**	**-0.36**	**0.13**	**-2.76**	**0.006**	**-0.62**	**-0.11**
	HWE (Yes)	-0.03	0.13	-0.27	0.789	-0.28	0.21
	Source of Control (Mixed)	-0.18	0.21	-0.86	0.388	-0.59	0.23
	Source of Control (PB)	-0.02	0.09	-0.23	0.821	-0.20	0.16
CC vs. CG + GG	Intercept	20.60	25.07	0.82	0.411	-28.53	69.74
	Year	-0.01	0.01	-0.80	0.421	-0.03	0.01
	Case_control_ratio CC vs. CG+GG	0.05	0.22	0.24	0.813	-0.38	0.48
	Cancer Type (Blood Cancer)	0.24	0.24	1.00	0.319	-0.23	0.71
	Cancer Type (Bone Cancer)	0.83	1.47	0.56	0.572	-2.05	3.70
	Cancer Type (Brain Cancer)	0.05	0.31	0.15	0.881	-0.56	0.65
	Cancer Type (Breast Cancer)	-0.15	0.21	-0.72	0.470	-0.56	0.26
	Cancer Type (Colon Cancer)	-0.36	0.26	-1.37	0.169	-0.86	0.15
	Cancer Type (Head and neck Cancer)	0.46	0.51	0.91	0.365	-0.54	1.46
	Cancer Type (Kidney Cancer)	0.57	0.47	1.21	0.225	-0.35	1.50
	Cancer Type (Laryngeal Cancer)	-0.08	0.33	-0.25	0.805	-0.73	0.56
	**Cancer Type (Liver Cancer)**	**-0.87**	**0.34**	**-2.53**	**0.012**	**-1.54**	**-0.19**
	Cancer Type (Lung Cancer)	-0.21	0.25	-0.81	0.419	-0.70	0.29
	Cancer Type (Nasopharyngeal Cancer)	0.48	0.28	1.74	0.082	-0.06	1.03
	Cancer Type (Ovarian Cancer)	-0.25	0.27	-0.92	0.357	-0.78	0.28
	Cancer Type (Prostate Cancer)	-0.31	0.47	-0.67	0.501	-1.23	0.60
	Cancer Type (Skin Cancer)	-0.02	0.29	-0.06	0.955	-0.58	0.55
	Cancer Type (Thyroid Cancer)	-0.36	0.51	-0.70	0.484	-1.36	0.64
	**Ethnicity (Caucasian)**	**-0.27**	**0.12**	**-2.21**	**0.027**	**-0.52**	**-0.03**
	Ethnicity (Mixed)	-0.38	0.19	-1.95	0.051	-0.76	0.00
	HWE (Yes)	-0.11	0.24	-0.46	0.643	-0.59	0.37
	Source of Control (Mixed)	-0.21	0.31	-0.67	0.503	-0.82	0.40
	Source of Control (PB)	-0.06	0.13	-0.48	0.634	-0.32	0.19
**rs1805794: Blood cancer subgroup**							
**Model**	**Variable**	**Estimate**	**SE**	**Zval**	**Pval**	**CI_lb**	**CI_ub**
C vs. G	Intercept	-10.60	198.43	-0.05	0.957	-399.51	378.31
	Year	0.01	0.10	0.06	0.955	-0.19	0.20
	Case_control_ratio C vs. G	0.11	0.90	0.12	0.904	-1.65	1.87
	**Ethnicity (Caucasian)**	**-1.07**	**0.23**	**-4.66**	**0.000**	**-1.52**	**-0.62**
	Ethnicity (Mixed)	-1.16	0.85	-1.37	0.171	-2.81	0.50

	HWE (Yes)	0.29	1.32	0.22	0.823	-2.29	2.88
	Source of Control (PB)	-0.01	0.25	-0.03	0.979	-0.49	0.48
CC vs. GG	Intercept	235.33	145.55	1.62	0.106	-49.95	520.60
	Year	-0.12	0.07	-1.61	0.107	-0.26	0.03
	Case_control_ratio CC vs. GG	1.15	0.64	1.81	0.071	-0.10	2.41
	**Ethnicity (Caucasian)**	**-1.49**	**0.22**	**-6.79**	**0.000**	**-1.92**	**-1.06**
	**Ethnicity (Mixed)**	**-2.58**	**0.67**	**-3.84**	**0.000**	**-3.90**	**-1.27**
	HWE (Yes)	-0.27	1.27	-0.21	0.832	-2.77	2.23
	Source of Control (PB)	-0.19	0.23	-0.81	0.418	-0.64	0.27
CG vs. GG	Intercept	26.55	166.68	0.16	0.873	-300.14	353.24
	Year	-0.01	0.08	-0.16	0.876	-0.18	0.15
	Case_control_ratio CG vs. GG	0.38	0.71	0.53	0.594	-1.01	1.76
	**Ethnicity (Caucasian)**	**-0.93**	**0.28**	**-3.35**	**0.001**	**-1.48**	**-0.39**
	Ethnicity (Mixed)	-1.19	0.81	-1.48	0.139	-2.78	0.39
	HWE (Yes)	-0.14	0.57	-0.24	0.810	-1.24	0.97
	Source of Control (PB)	0.12	0.25	0.47	0.642	-0.37	0.60
CC + CG vs. GG	Intercept	75.29	130.51	0.58	0.564	-180.50	331.07
	Year	-0.04	0.06	-0.57	0.568	-0.16	0.09
	Case_control_ratio CC+CG vs. GG	0.56	0.54	1.03	0.301	-0.50	1.63
	**Ethnicity (Caucasian)**	**-1.10**	**0.21**	**-5.27**	**0.000**	**-1.51**	**-0.69**
	**Ethnicity (Mixed)**	**-1.54**	**0.57**	**-2.69**	**0.007**	**-2.67**	**-0.42**
	HWE (Yes)	-0.22	0.46	-0.48	0.631	-1.12	0.68
	Source of Control (PB)	0.03	0.21	0.16	0.875	-0.37	0.44
CC vs. CG + GG	Intercept	363.82	161.34	2.25	0.024	47.60	680.04
	Year	-0.18	0.08	-2.26	0.024	-0.34	-0.02
	Case_control_ratio CC vs. CG+GG	1.56	0.74	2.10	0.036	0.11	3.02
	**Ethnicity (Caucasian)**	**-0.92**	**0.17**	**-5.27**	**0.000**	**-1.26**	**-0.58**
	**Ethnicity (Mixed)**	**-2.36**	**0.69**	**-3.43**	**0.001**	**-3.71**	**-1.01**
	HWE (Yes)	0.22	0.69	0.32	0.749	-1.14	1.58
	Source of Control (PB)	-0.26	0.18	-1.42	0.156	-0.62	0.10
**rs1805794: Asian subgroup**							
**Model**	**Variable**	**Estimate**	**SE**	**Zval**	**Pval**	**CI_lb**	**CI_ub**
C vs. G	Intercept	-49.57	80.63	-0.61	0.539	-207.60	108.47
	Year	0.03	0.04	0.64	0.524	-0.05	0.10
	Case_control_ratio C vs. G	-1.70	1.03	-1.66	0.098	-3.71	0.31
	Cancer Type (Respiratory/Urinary Cancer)	0.08	0.34	0.24	0.807	-0.59	0.75
	HWE (Yes)	-0.25	0.59	-0.43	0.668	-1.42	0.91
	Source of Control (PB)	0.05	0.37	0.14	0.891	-0.67	0.77
CC vs. GG	Intercept	-72.13	124.11	-0.58	0.561	-315.38	171.12
	Year	0.04	0.06	0.60	0.548	-0.08	0.16
	Case_control_ratio CC vs. GG	-2.35	1.64	-1.43	0.152	-5.56	0.87
	Cancer Type (Respiratory/Urinary Cancer)	0.34	0.54	0.63	0.530	-0.73	1.41

	HWE (Yes)	-0.08	0.90	-0.08	0.933	-1.85	1.70
	Source of Control (PB)	-0.23	0.57	-0.40	0.686	-1.36	0.89
CG vs. GG	Intercept	-29.92	41.57	-0.72	0.472	-111.39	51.55
	Year	0.02	0.02	0.75	0.455	-0.03	0.06
	**case_control_ratio_CGvs GG**	**-1.27**	**0.39**	**-3.26**	**0.001**	**-2.03**	**-0.51**
	Cancer Type (Respiratory/Urinary Cancer)	-0.06	0.18	-0.34	0.733	-0.41	0.29
	HWE (Yes)	0.01	0.26	0.04	0.969	-0.49	0.51
	Source of Control (PB)	0.15	0.19	0.79	0.430	-0.22	0.51
CC + CG vs. GG	Intercept	-52.29	78.64	-0.66	0.506	-206.41	101.84
	Year	0.03	0.04	0.68	0.496	-0.05	0.10
	Case_control_ratio CC+CG vs. GG	-1.27	0.90	-1.41	0.157	-3.04	0.49
	Cancer Type (Respiratory/Urinary Cancer)	0.06	0.34	0.19	0.848	-0.60	0.73
	HWE (Yes)	0.04	0.49	0.09	0.927	-0.91	1.00
	Source of Control (PB)	0.01	0.36	0.02	0.985	-0.71	0.72
CC vs. CG + GG	Intercept	-27.48	78.15	-0.35	0.725	-180.66	125.69
	Year	0.01	0.04	0.38	0.706	-0.06	0.09
	**Case_control_ratio CC vs. CG+GG**	**-2.04**	**1.03**	**-1.98**	**0.047**	**-4.06**	**-0.02**
	Cancer Type (Respiratory/Urinary Cancer)	0.35	0.35	1.00	0.318	-0.34	1.04
	HWE (Yes)	-0.07	0.56	-0.12	0.902	-1.17	1.03
	Source of Control (PB)	-0.13	0.38	-0.34	0.736	-0.86	0.61

**rs1805794: HB subgroup**							
**Model**	**Variable**	**Estimate**	**SE**	**Zval**	**Pval**	**CI_lb**	**CI_ub**
C vs. G	Intercept	26.48	44.29	0.60	0.550	-60.33	113.28
	Year	-0.01	0.02	-0.58	0.559	-0.06	0.03
	Case_control_ratio C vs. G	0.01	0.39	0.02	0.987	-0.76	0.77
	**Ethnicity (Caucasian)**	**-0.35**	**0.15**	**-2.26**	**0.024**	**-0.65**	**-0.05**
	Ethnicity (Mixed)	0.46	0.75	0.61	0.540	-1.01	1.93
	HWE (Yes)	-0.21	0.22	-0.95	0.343	-0.65	0.23
	Cancer Type (Blood Cancer)	0.23	0.30	0.76	0.447	-0.36	0.82
	Cancer Type (Bone Cancer)	-0.50	1.48	-0.34	0.737	-3.39	2.40
	Cancer Type (Brain Cancer)	-0.14	0.28	-0.51	0.611	-0.69	0.41
	Cancer Type (Breast Cancer)	-0.30	0.24	-1.25	0.210	-0.78	0.17
	Cancer Type (Colon Cancer)	-0.17	0.24	-0.72	0.473	-0.64	0.30
	Cancer Type (Kidney Cancer)	-0.17	0.42	-0.40	0.688	-0.98	0.65
	Cancer Type (Laryngeal Cancer)	-0.28	0.31	-0.90	0.370	-0.90	0.33
	**Cancer Type (Liver Cancer)**	**-1.02**	**0.26**	**-3.92**	**0.000**	**-1.54**	**-0.51**
	Cancer Type (Lung Cancer)	-0.17	0.28	-0.61	0.543	-0.72	0.38
	Cancer Type (Nasopharyngeal Cancer)	0.35	0.25	1.40	0.160	-0.14	0.84
	Cancer Type (Skin Cancer)	-0.02	0.25	-0.10	0.923	-0.51	0.47
	Cancer Type (Thyroid Cancer)	-0.07	0.52	-0.14	0.891	-1.10	0.96
CC vs. GG	Intercept	80.78	59.70	1.35	0.176	-36.24	197.80
	Year	-0.04	0.03	-1.34	0.179	-0.10	0.02

	Case_control_ratio CC vs. GG	0.58	0.47	1.24	0.216	-0.34	1.51
	**Ethnicity (Caucasian)**	**-0.62**	**0.20**	**-3.19**	**0.001**	**-1.01**	**-0.24**
	Ethnicity (Mixed)	0.02	0.76	0.02	0.983	-1.48	1.52
	HWE (Yes)	-0.51	0.32	-1.60	0.110	-1.14	0.12
	**Cancer Type (Blood Cancer)**	**0.86**	**0.39**	**2.18**	**0.029**	**0.09**	**1.62**
	Cancer Type (Bone Cancer)	0.68	1.52	0.45	0.652	-2.29	3.66
	Cancer Type (Brain Cancer)	-0.17	0.43	-0.40	0.687	-1.02	0.67
	Cancer Type (Breast Cancer)	-0.26	0.32	-0.82	0.412	-0.89	0.36
	Cancer Type (Colon Cancer)	-0.27	0.36	-0.75	0.455	-0.98	0.44
	Cancer Type (Kidney Cancer)	0.21	0.56	0.37	0.712	-0.89	1.31
	Cancer Type (Laryngeal Cancer)	-0.14	0.45	-0.31	0.757	-1.03	0.75
	**Cancer Type (Liver Cancer)**	**-1.41**	**0.47**	**-3.03**	**0.002**	**-2.33**	**-0.50**
	Cancer Type (Lung Cancer)	-0.07	0.38	-0.19	0.850	-0.82	0.68
	Cancer Type (Nasopharyngeal Cancer)	0.73	0.39	1.86	0.062	-0.04	1.50
	Cancer Type (Skin Cancer)	0.14	0.36	0.38	0.701	-0.56	0.84
	Cancer Type (Thyroid Cancer)	0.02	0.59	0.03	0.979	-1.14	1.17
CG vs. GG	Intercept	2.33	33.08	0.07	0.944	-62.51	67.18
	Year	0.00	0.02	-0.06	0.954	-0.03	0.03
	Case_control_ratio CG vs. GG	0.13	0.28	0.45	0.651	-0.42	0.67
	**Ethnicity (Caucasian)**	**-0.29**	**0.12**	**-2.46**	**0.014**	**-0.52**	**-0.06**
	Ethnicity (Mixed)	0.63	0.43	1.45	0.146	-0.22	1.48
	HWE (Yes)	-0.14	0.17	-0.79	0.430	-0.47	0.20
	Cancer Type (Blood Cancer)	-0.03	0.24	-0.10	0.917	-0.51	0.45
	Cancer Type (Bone Cancer)	-0.31	0.33	-0.95	0.343	-0.95	0.33
	Cancer Type (Brain Cancer)	-0.27	0.21	-1.25	0.212	-0.69	0.15
	Cancer Type (Breast Cancer)	-0.32	0.18	-1.80	0.072	-0.66	0.03
	Cancer Type (Colon Cancer)	-0.16	0.19	-0.84	0.399	-0.53	0.21
	Cancer Type (Kidney Cancer)	-0.27	0.29	-0.91	0.360	-0.84	0.31
	Cancer Type (Laryngeal Cancer)	-0.40	0.24	-1.68	0.092	-0.86	0.07
	**Cancer Type (Liver Cancer)**	**-0.94**	**0.26**	**-3.62**	**0.000**	**-1.45**	**-0.43**
	Cancer Type (Lung Cancer)	-0.14	0.20	-0.69	0.490	-0.54	0.26
	Cancer Type (Nasopharyngeal Cancer)	0.12	0.21	0.58	0.560	-0.29	0.54
	Cancer Type (Skin Cancer)	-0.02	0.19	-0.08	0.935	-0.39	0.36
	Cancer Type (Thyroid Cancer)	0.01	0.32	0.02	0.987	-0.63	0.64
CC + CG vs. GG	Intercept	29.28	28.98	1.01	0.312	-27.52	86.08
	Year	-0.01	0.01	-0.99	0.321	-0.04	0.01
	Case_control_ratio CC+CG vs. GG	0.13	0.23	0.58	0.565	-0.32	0.59
	**Ethnicity (Caucasian)**	**-0.36**	**0.10**	**-3.77**	**0.000**	**-0.55**	**-0.17**
	Ethnicity (Mixed)	0.53	0.39	1.36	0.175	-0.23	1.29
	HWE (Yes)	-0.24	0.14	-1.67	0.095	-0.52	0.04
	Cancer Type (Blood Cancer)	0.19	0.20	0.96	0.337	-0.20	0.59
	Cancer Type (Bone Cancer)	-0.33	0.29	-1.13	0.257	-0.91	0.24

	Cancer Type (Brain Cancer)	-0.16	0.18	-0.90	0.367	-0.52	0.19
	**Cancer Type (Breast Cancer)**	**-0.35**	**0.15**	**-2.36**	**0.018**	**-0.64**	**-0.06**
	Cancer Type (Colon Cancer)	-0.16	0.16	-0.97	0.331	-0.47	0.16
	Cancer Type (Kidney Cancer)	-0.19	0.24	-0.81	0.420	-0.65	0.27
	Cancer Type (Laryngeal Cancer)	-0.29	0.20	-1.43	0.154	-0.68	0.11
	**Cancer Type (Liver Cancer)**	**-1.03**	**0.19**	**-5.40**	**0.000**	**-1.40**	**-0.66**
	Cancer Type (Lung Cancer)	-0.14	0.17	-0.82	0.410	-0.46	0.19
	**Cancer Type (Nasopharyngeal Cancer)**	**0.36**	**0.17**	**2.11**	**0.035**	**0.03**	**0.69**
	Cancer Type (Skin Cancer)	-0.01	0.16	-0.04	0.972	-0.33	0.32
	Cancer Type (Thyroid Cancer)	-0.01	0.28	-0.03	0.976	-0.56	0.54
CC vs. CG + GG	**Intercept**	**132.85**	**65.84**	**2.02**	**0.044**	**3.81**	**261.89**
	**Year**	**-0.07**	**0.03**	**-2.02**	**0.044**	**-0.13**	**0.00**
	Case_control_ratio CC vs. CG+GG	0.28	0.54	0.52	0.603	-0.77	1.33
	Ethnicity (Caucasian)	-0.44	0.24	-1.85	0.064	-0.91	0.03
	Ethnicity (Mixed)	-0.34	0.83	-0.41	0.681	-1.96	1.28
	HWE (Yes)	-0.18	0.39	-0.47	0.639	-0.96	0.59
	Cancer Type (Blood Cancer)	0.71	0.49	1.45	0.146	-0.25	1.68
	Cancer Type (Bone Cancer)	1.45	1.58	0.92	0.359	-1.64	4.54
	Cancer Type (Brain Cancer)	0.42	0.50	0.83	0.407	-0.57	1.40
	Cancer Type (Breast Cancer)	0.02	0.40	0.05	0.964	-0.77	0.81
	Cancer Type (Colon Cancer)	0.01	0.46	0.02	0.980	-0.90	0.92
	Cancer Type (Kidney Cancer)	0.80	0.69	1.16	0.244	-0.55	2.15
	Cancer Type (Laryngeal Cancer)	0.40	0.54	0.74	0.458	-0.65	1.45
	Cancer Type (Liver Cancer)	-0.57	0.60	-0.94	0.345	-1.74	0.61
	Cancer Type (Lung Cancer)	0.05	0.48	0.10	0.923	-0.89	0.99
	Cancer Type (Nasopharyngeal Cancer)	0.92	0.49	1.86	0.063	-0.05	1.88
	Cancer Type (Skin Cancer)	0.14	0.45	0.31	0.758	-0.75	1.03
	Cancer Type (Thyroid Cancer)	0.05	0.69	0.07	0.947	-1.31	1.40
**rs1805794: PB subgroup**							
**Model**	**Variable**	**Estimate**	**SE**	**Zval**	**Pval**	**CI_lb**	**CI_ub**
C vs. G	Intercept	-16.50	36.74	-0.45	0.653	-88.50	55.50
	Year	0.01	0.02	0.47	0.640	-0.03	0.04
	Case_control_ratio C vs. G	0.13	0.25	0.50	0.618	-0.37	0.62
	Ethnicity (Caucasian)	-0.34	0.19	-1.85	0.065	-0.71	0.02
	Ethnicity (Mixed)	-0.44	0.23	-1.89	0.059	-0.89	0.02
	HWE (Yes)	-0.18	1.18	-0.15	0.877	-2.49	2.12
	Cancer Type (Blood Cancer)	-0.11	0.32	-0.34	0.736	-0.73	0.52
	Cancer Type (Breast Cancer)	-0.24	0.28	-0.84	0.401	-0.79	0.32
	Cancer Type (Colon Cancer)	-0.33	0.52	-0.65	0.516	-1.35	0.68
	Cancer Type (Head and neck Cancer)	-0.15	0.56	-0.26	0.796	-1.25	0.96
	Cancer Type (Lung Cancer)	-0.67	0.39	-1.71	0.088	-1.43	0.10
	Cancer Type (Ovarian Cancer)	-0.20	0.32	-0.63	0.527	-0.82	0.42

	Cancer Type (Prostate Cancer)	0.01	1.24	0.01	0.994	-2.43	2.45
CC vs. GG	Intercept	-36.51	43.03	-0.85	0.396	-120.84	47.83
	Year	0.02	0.02	0.86	0.387	-0.02	0.06
	Case_control_ratio CC vs. GG	0.15	0.30	0.49	0.621	-0.45	0.75
	Ethnicity (Caucasian)	-0.33	0.25	-1.32	0.187	-0.81	0.16
	Ethnicity (Mixed)	-0.50	0.31	-1.60	0.109	-1.11	0.11
	HWE (Yes)	-0.12	1.19	-0.10	0.921	-2.45	2.21
	Cancer Type (Blood Cancer)	-0.06	0.41	-0.15	0.878	-0.87	0.75
	Cancer Type (Breast Cancer)	-0.38	0.38	-1.00	0.317	-1.12	0.36
	Cancer Type (Colon Cancer)	-0.67	0.60	-1.12	0.264	-1.84	0.51
	Cancer Type (Head and neck Cancer)	0.11	0.63	0.17	0.866	-1.13	1.35
	Cancer Type (Lung Cancer)	-0.86	0.48	-1.80	0.071	-1.80	0.07
	Cancer Type (Ovarian Cancer)	-0.42	0.42	-1.00	0.318	-1.23	0.40
	Cancer Type (Prostate Cancer)	-0.23	1.29	-0.17	0.861	-2.75	2.30
CG vs. GG	Intercept	13.13	29.73	0.44	0.659	-45.15	71.40
	Year	-0.01	0.01	-0.42	0.672	-0.04	0.02
	Case_control_ratio CG vs. GG	0.03	0.19	0.13	0.897	-0.35	0.40
	Ethnicity (Caucasian)	-0.30	0.17	-1.77	0.077	-0.62	0.03
	Ethnicity (Mixed)	-0.36	0.20	-1.82	0.069	-0.76	0.03
	HWE (Yes)	-0.11	0.20	-0.54	0.587	-0.51	0.29
	Cancer Type (Blood Cancer)	-0.21	0.25	-0.86	0.388	-0.70	0.27
	Cancer Type (Breast Cancer)	-0.20	0.22	-0.92	0.357	-0.63	0.23
	Cancer Type (Colon Cancer)	-0.21	0.37	-0.58	0.563	-0.93	0.51
	Cancer Type (Head and neck Cancer)	-0.18	0.41	-0.45	0.654	-0.98	0.61
	Cancer Type (Lung Cancer)	-0.28	0.28	-1.00	0.318	-0.82	0.27
	Cancer Type (Ovarian Cancer)	-0.20	0.24	-0.82	0.410	-0.68	0.28
	Cancer Type (Prostate Cancer)	0.17	0.36	0.48	0.634	-0.54	0.88
CC + CG vs. GG	Intercept	2.14	30.92	0.07	0.945	-58.47	62.75
	Year	0.00	0.02	-0.05	0.958	-0.03	0.03
	Case_control_ratio CC+CG vs. GG	0.11	0.22	0.47	0.635	-0.33	0.54
	Ethnicity (Caucasian)	-0.30	0.19	-1.59	0.111	-0.68	0.07
	Ethnicity (Mixed)	-0.43	0.23	-1.89	0.059	-0.87	0.02
	HWE (Yes)	-0.10	0.23	-0.46	0.644	-0.55	0.34
	Cancer Type (Blood Cancer)	-0.16	0.29	-0.55	0.584	-0.73	0.41
	Cancer Type (Breast Cancer)	-0.22	0.26	-0.87	0.382	-0.73	0.28
	Cancer Type (Colon Cancer)	-0.30	0.41	-0.73	0.463	-1.10	0.50
	Cancer Type (Head and neck Cancer)	-0.11	0.43	-0.25	0.800	-0.96	0.74
	Cancer Type (Lung Cancer)	-0.36	0.32	-1.13	0.260	-0.98	0.26
	Cancer Type (Ovarian Cancer)	-0.23	0.29	-0.80	0.424	-0.79	0.33
	Cancer Type (Prostate Cancer)	0.11	0.41	0.28	0.781	-0.69	0.92
CC vs. CG + GG	Intercept	-31.36	29.82	-1.05	0.293	-89.81	27.09
	Year	0.02	0.01	1.06	0.288	-0.01	0.04

	Case_control_ratio CC vs. CG+GG	0.08	0.26	0.30	0.767	-0.43	0.58
	Ethnicity (Caucasian)	-0.15	0.17	-0.86	0.390	-0.49	0.19
	Ethnicity (Mixed)	-0.32	0.21	-1.49	0.136	-0.74	0.10
	HWE (Yes)	0.03	1.17	0.02	0.982	-2.27	2.32
	Cancer Type (Blood Cancer)	0.06	0.32	0.19	0.852	-0.57	0.69
	Cancer Type (Breast Cancer)	-0.25	0.29	-0.86	0.391	-0.82	0.32
	Cancer Type (Colon Cancer)	-0.53	0.49	-1.09	0.278	-1.49	0.43
	Cancer Type (Head and neck Cancer)	0.24	0.52	0.47	0.640	-0.77	1.26
	Cancer Type (Lung Cancer)	-0.51	0.35	-1.43	0.151	-1.20	0.19
	Cancer Type (Ovarian Cancer)	-0.33	0.32	-1.04	0.299	-0.96	0.30
	Cancer Type (Prostate Cancer)	-0.22	1.23	-0.18	0.856	-2.64	2.20

Bold indicates the statistically significant sources of heterogeneity under specific subgroups and genetic models.

Table S3. Publication bias for NBN gene polymorphisms was assessed using Begg’s and Egger’s tests.

Polymorphisms	Begg's test			Egger's test			
	**z-value**	**p-value**	**Comment**	**t-value**	**d.f.**	**p-value**	**Comment**
**rs1805794**							
CC vs. GG	-0.0784	0.9374	No publication bias	-0.6587	57	0.5127	No publication bias
C vs. G	-0.2092	0.8342	No publication bias	-0.4565	57	0.6494	No publication bias
**rs709816**							
CC vs. TT	1.23	0.2171	No publication bias	2.2343	10	0.0223	Publication bias
C vs. T	0.8229	0.4106	No publication bias	2.7327	10	0.0211	Publication bias

For testing the publication bias we used the Begg’s and Egger’s test with the following hypothesis:
H0 (Null hypothesis): Symmetry in the funnel plot vs.
H1 (Alternative hypothesis): Asymmetry in the funnel plot
If p-value ≤ 0.05we reject Null hypothesis (H0)
If p-value > 0.05 we accept the null hypothesis (H0) and reject the alternative hypothesis (H1).

Table S4

Results of false positive report probability analysis for significant findings of the meta-analysis.

Genotype and Variables	OR (95% CI)	Statistical Power[a]	FPRP values for prior probabilities at					
			0.25	0.1	0.01	0.001	0.0001	0.00001
rs1805794 and Bladder cancer								
CC vs. GG	1.09 [1.01; 1.16]	1.000	0.020[b]	0.056[b]	0.397	0.869	0.985	0.998
CC + CG vs. GG	1.13 [1.02; 1.24]	1.000	0.029[b]	0.082[b]	0.495	0.908	0.990	0.999
CG vs. GG	1.13 [1.03; 1.24]	1.000	0.029[b]	0.082[b]	0.495	0.908	0.990	0.999
rs1805794 and Nasopharyngeal cancer								
CC vs. GG	1.56 [1.10; 2.20]	0.412	0.076[b]	0.197[b]	0.730	0.965	0.996	1.000
CC vs. CG + GG	2.28 [1.06; 4.89]	0.141	0.421	0.686	0.960	0.996	1.000	1.000
CC + CG vs. GG	1.58 [1.32; 1.90]	0.290	0.000[b]	0.000[b]	0.000[b]	0.004[b]	0.039[b]	0.287
CG vs. GG	1.98 [1.66; 2.35]	0.001	0.000[b]	0.000[b]	0.000[b]	0.000[b]	0.000[b]	0.001[b]
rs1805794 and Brain cancer								
C vs. G	1.49 [1.20; 1.84]	0.525	0.001[b]	0.004[b]	0.038[b]	0.287	0.801	0.976
rs1805794 and HB population								
CC vs. GG	1.11 [1.01; 1.23]	1.000	0.122[b]	0.294	0.821	0.979	0.998	1.000
CC vs. CC + GG	1.27 [1.01; 1.60]	0.921	0.122[b]	0.294	0.821	0.979	0.998	1.000
rs709816 and Overall cancer								
CC vs. TT	0.95 [0.89; 1.00]	1.000	0.130[b]	0.310	0.832	0.980	0.998	1.000
rs709816 and Breast cancer								
CC vs. TT	0.88 [0.77; 0.99]	1.000	0.091[b]	0.231	0.768	0.971	0.997	1.000
rs709816 and HB population								
CC vs. CT + TT	0.79 [0.62; 1.00]	0.915	0.141[b]	0.330	0.844	0.982	0.998	1.000

[a]Statistical power was calculated using the number of observations in each subgroup and the corresponding ORs and *P* values in this table.

[b]The level of false-positive report probability threshold was set at 0.2 and noteworthy findings are presented

Table S5. Significantly enriched disease/cancer terms in the DisGeNET database by the multiple cancer-causing meta-gene-set including the NBN gene.

Disease terms	Cancer type	P-value	Adjusted P-value	Genes
Meningioma	Ovarian cancer	2.88E-09	3.01E-06	IL6;TERT;ADIPOQ;BRCA1;IGF;**NBN**
Breast Cancer, Familial Male	Breast Cancer	1.20E-08	4.58E-06	NBN;BRCA1;PALB2
Malignant neoplasm of endometrium	Endometrial cancer	1.50E-08	4.58E-06	IL6;TERT;ADIPOQ;**NBN**;BRCA1;IGF1
Uterine Corpus Cancer	Uterine cancer	1.53E-08	4.58E-06	IL6;TERT;ADIPOQ;**NBN**;BRCA1;IGF1
bilateral breast cancer	Breast Cancer	8.47E-08	1.56E-05	**NBN**;BRCA1;PALB2
Hereditary Nonpolyposis Colorectal Cancer	Colon Cancer	1.10E-07	1.64E-05	**NBN**;BRCA1;IGF1;PALB2
Prostatic Neoplasms	Prostate cancer	1.26E-07	1.76E-05	IL6;TERT;ADIPOQ;NBN;BRCA1;IGF1;PALB2
Endometrial Carcinoma	Uterine cancer	1.40E-07	1.84E-05	IL6;TERT;ADIPOQ;**NBN;**BRCA1;IGF1
Primary peritoneal carcinoma	Ovarian cancer	1.53E-07	1.88E-05	**NBN;**BRCA1;PALB2
Breast Cancer, Familial	Breast Cancer	2.31E-07	2.49E-05	**NBN**;BRCA1;IGF1;PALB2
Leukemia, Myelocytic, Acute	Blood Cancer	2.38E-07	2.49E-05	IL6;TERT;ADIPOQ;**NBN;**BRCA1;IGF1;PALB2
ovarian neoplasm	Ovarian cancer	2.70E-07	2.63E-05	IL6;TERT;**NBN**;BRCA1;IGF1;PALB2
Laryngeal neoplasm	Laryngeal Cancer	4.45E-07	3.88E-05	TERT;**NBN;**BRCA1
Colorectal Neoplasms	Colon Cancer	6.00E-07	4.14E-05	IL6;TERT;ADIPOQ;**NBN;**BRCA1;IGF1
Lymphoma, Non-Hodgkin	Blood Cancer	7.31E-07	4.50E-05	IL6;TERT;ADIPOQ;**NBN;**BRCA1
Hematologic Neoplasms	Blood Cancer	8.60E-07	5.15E-05	IL6;TERT;**NBN**;BRCA1;PALB2
Hereditary Breast and Ovarian Cancer Syndrome	Breast and ovarian Cancer	9.20E-07	5.35E-05	**NBN;**BRCA1;PALB2
Prostate cancer, familial	Prostate cancer	9.75E-07	5.52E-05	**NBN;**BRCA1;PALB2
Medulloblastoma	Brain cancer	1.14E-06	5.99E-05	IL6;TERT;**NBN;**IGF1;PALB2
Renal Cell Carcinoma	Kidney cancer	2.31E-06	8.63E-05	IL6;TERT;ADIPOQ;**NBN;**BRCA1;IGF1
Stomach Carcinoma	Stomach Cancer	2.39E-06	8.63E-05	IL6;TERT;ADIPOQ;ACYP2;**NBN;**BRCA1;IGF1
Nasopharyngeal carcinoma	Nasopharyngeal cancer	4.58E-06	1.25E-04	IL6;TERT;**NBN;**BRCA1;IGF1
Carcinoma breast stage IV	Breast cancer	9.56E-06	2.04E-04	IL6;NBN;BRCA1;IGF1
Squamous cell carcinoma of the head and neck	Skin cancer	1.09E-05	2.16E-04	IL6;TERT**;NBN**;BRCA1;IGF1
Childhood Acute Lymphoblastic Leukemia	Blood Cancer	1.55E-05	2.73E-04	IL6;TERT**;NBN**;IGF1

Prostate carcinoma	Prostate cancer	1.63E-05	2.78E-04	IL6;TERT;ADIPOQ;**NBN;**BRCA1;IGF1;PALB2
leukemia	Blood Cancer	1.95E-05	3.15E-04	IL6;TERT;ADIPOQ;**NBN;**BRCA1;IGF1
Malignant neoplasm of prostate	Prostate cancer	2.00E-05	3.19E-04	IL6;TERT;ADIPOQ;**NBN;**BRCA1;IGF1;PALB2
Malignant neoplasm of urinary bladder	Bladder cancer	2.94E-05	4.22E-04	IL6;TERT;**NBN**;BRCA1;IGF1
Carcinoma of bladder	Bladder cancer	3.17E-05	4.43E-04	IL6;TERT;**NBN;**BRCA1;IGF1
Lung Neoplasms	Lung Cancer	3.37E-05	4.62E-04	IL6;TERT;ADIPOQ;**NBN;**IGF1
Carcinoma of larynx	Laryngeal Cancer	3.69E-05	4.87E-04	**NBN;**BRCA1;IGF1
Bladder Neoplasm	Bladder cancer	3.97E-05	5.16E-04	IL6;TERT**;NBN**;BRCA1;IGF1
Ovarian Carcinoma	Ovarian cancer	4.08E-05	5.28E-04	IL6;TERT;**NBN;**BRCA1;IGF1;PALB2
Non-Small Cell Lung Carcinoma	Lung Cancer	4.53E-05	5.68E-04	IL6;TERT;ADIPOQ;**NBN;**BRCA1;IGF1
Primary malignant neoplasm of lung	Lung Cancer	4.83E-05	6.01E-04	IL6;TERT;ADIPOQ;**NBN;**BRCA1;IGF1
Lymphoma	Blood Cancer	5.61E-05	6.67E-04	IL6;TERT;**NBN;**BRCA1;IGF1
Multiple Myeloma	Blood Cancer	5.71E-05	6.68E-04	IL6;TERT**;NBN;**BRCA1;IGF1
Malignant neoplasm of lung	Lung Cancer	7.53E-05	7.81E-04	IL6;TERT;ADIPOQ;**NBN;**BRCA1;IGF1
melanoma	Skin cancer	7.62E-05	7.86E-04	IL6;TERT;**NBN**;BRCA1;IGF1;PALB2
Promyelocytic leukemia	Blood Cancer	8.37E-05	7.97E-04	TERT;**NBN**;BRCA1
Astrocytoma	Brain cancer	1.16E-04	9.89E-04	IL6;TERT;**NBN**;IGF1
Colorectal Carcinoma	Colon Cancer	2.11E-04	0.001465	IL6;TERT;ADIPOQ;**NBN;**BRCA1;IGF1
Squamous cell carcinoma of mouth	Oral Cancer	2.37E-04	0.0015408	TERT;**NBN**
Acute lymphocytic leukemia	Blood and Bone cancer	2.54E-04	0.0016176	IL6;TERT;**NBN**;IGF1
Squamous cell carcinoma	Skin cancer	3.17E-04	0.0019385	IL6;TERT**;NBN**;BRCA1;IGF1
Breast Carcinoma	Breast cancer	3.62E-04	0.0021258	IL6;TERT;ADIPOQ;**NBN;**BRCA1;IGF1;PALB2
Colorectal Cancer	Colon Cancer	4.14E-04	0.0023195	IL6;TERT;ADIPOQ;**NBN;**BRCA1;IGF1
Liver carcinoma	Liver Cancer	6.72E-04	0.0033365	IL6;TERT;ADIPOQ;**NBN;**BRCA1;IGF1
Acute leukemia	Blood and Bone cancer	7.56E-04	0.0036391	IL6;**NBN**;BRCA1
Hypopharyngeal Cancer	Head and neck cancer	0.003595	0.0104168	**NBN**

Table S6. Significantly enriched GO functions and KEGG pathways were identified in different databases for the multiple cancer-causing meta-gene set, including the NBN gene (p-value < 0.05), supported by a literature review.

Biological Process (BPs)		
**GO ID**	**GO Terms**	**Databases**
GO:0000724[1]	double-strand break repair via homologous recombination	GeneCodis, DEVID, Enrichr, ToppFun
GO:0000729[2]	DNA double-strand break processing	GeneCodis, DEVID, Enrichr, ToppFun
GO:0000723[3]	telomere maintenance	GeneCodis, DEVID,Enricher
GO:0006303[4]	double-strand break repair via nonhomologous end joining	GeneCodis, DEVID, Enrichr,ToppFun
GO:0006302[5]	double-strand break repair	GeneCodis, DEVID,Enrichr,ToppFun
GO:1901796[6]	regulation of signal transduction by p53 class mediator	GeneCodis, ToppFun
GO:0006260[7]	DNA replication	GeneCodis, DEVID,ToppFun
**Cellular Components (CCs)**		
**GO ID**	**GO Terms**	**Databases**
GO:0016605[8]	PML body	GeneCodis, DEVID,ToppFun
GO:0000781[9]	chromosome, telomeric region	GeneCodis, DEVID,ToppFun
GO:0005654[10]	nucleoplasm	GeneCodis, DEVID
GO:0030870[11]	Mre11 complex	GeneCodis, ToppFun
**Molecular Functions (MFs)**		
**GO ID**	**GO Terms**	**Databases**
GO:0005515[12]	protein binding	GeneCodis, DEVID
GO:0003684[13]	damaged DNA binding	GeneCodis, DEVID,ToppFun
GO:0047485[14]	protein N-terminus binding	GeneCodis, ToppFun
**KEGG**		
**Annotation ID**	**Pathway**	**Other Enriched Databases**
hsa03440[15]	Homologous recombination	GeneCodis, Enrichr
hsa04218[16]	Cellular senescence	GeneCodis, Enrichr

Supplementary figures

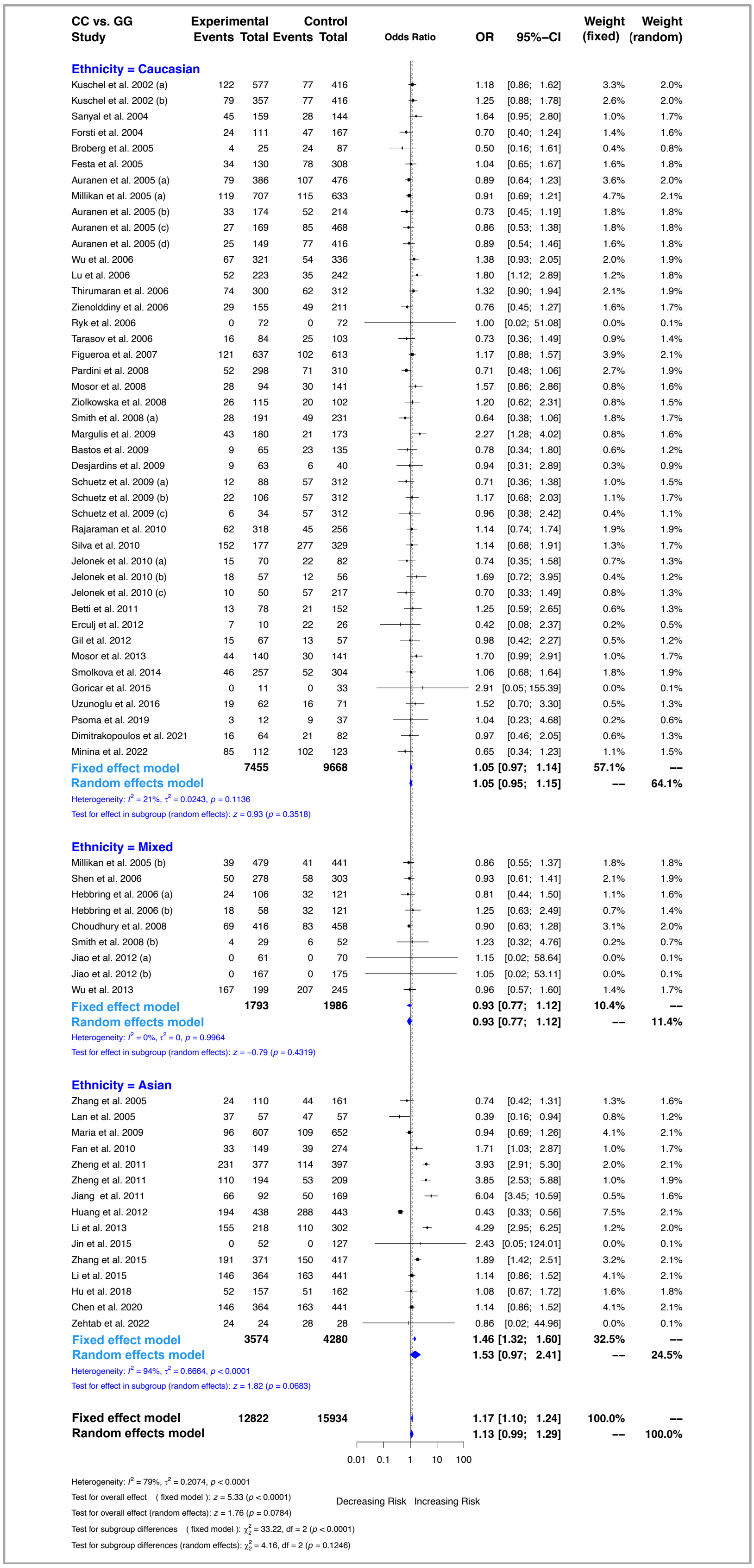

Figure S1. The association between rs1805794 polymorphism and cancer risk by showing the forest plot under homozygote comparison (CC vs. GG).

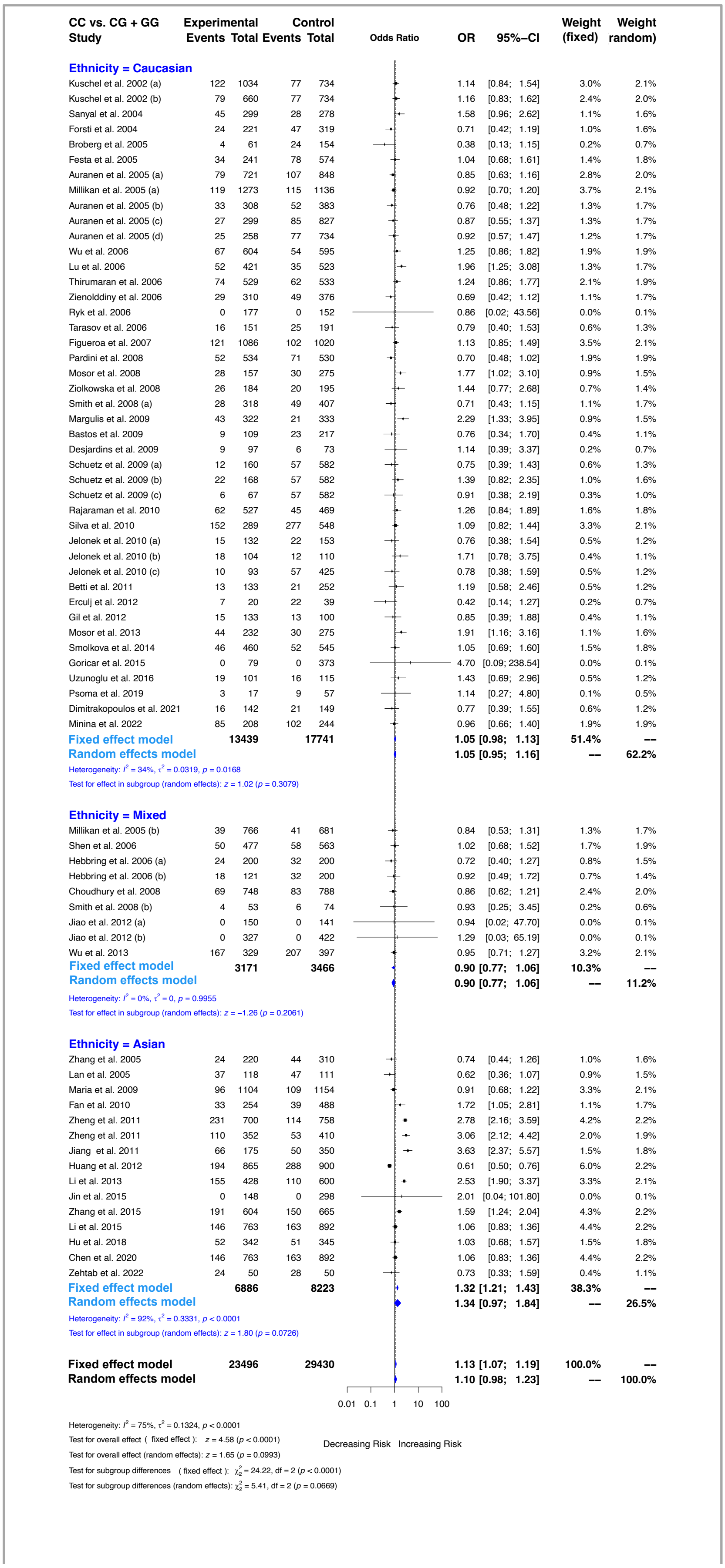

Figure S2. The association between rs1805794 polymorphism and cancer risk by showing the forest plot under recessive comparison (CC vs. CG + GG).

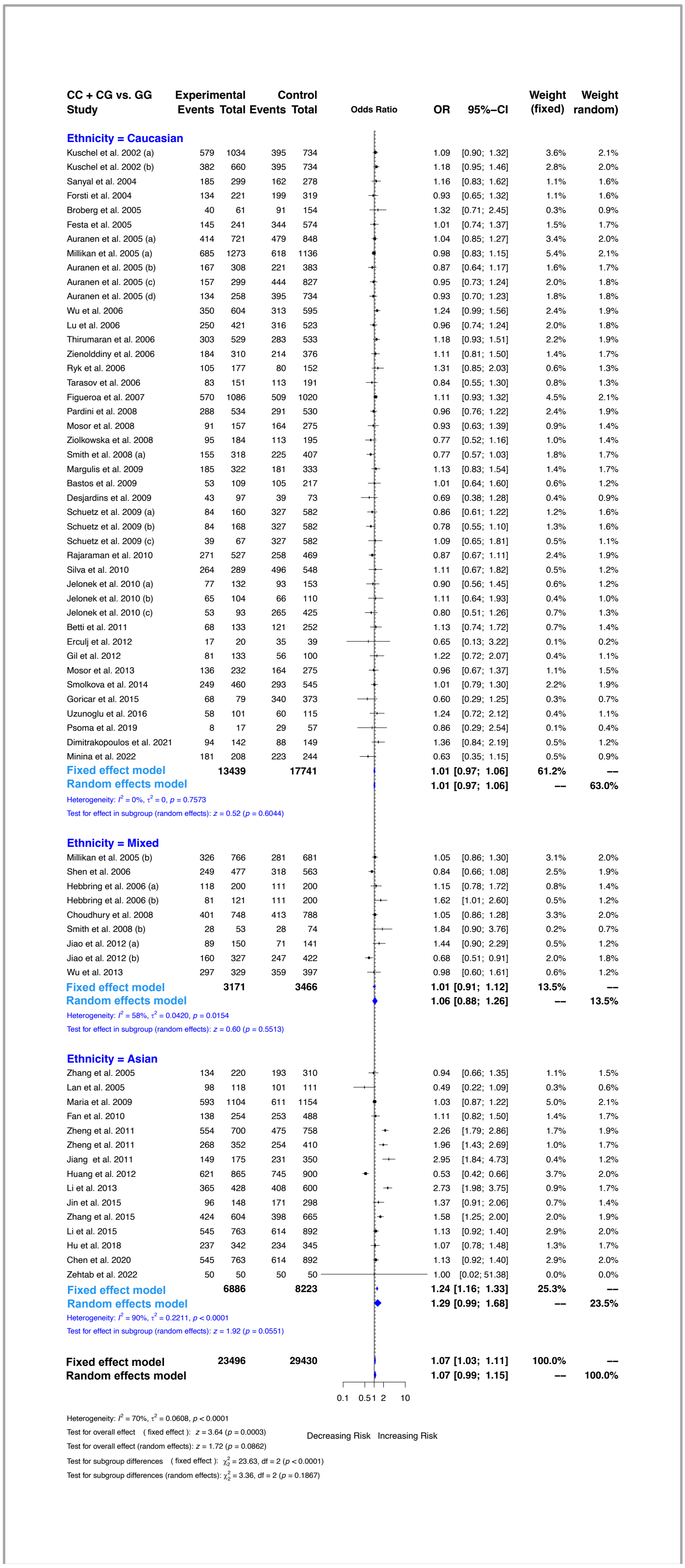

Figure S3. The association between rs1805794 polymorphism and cancer risk by showing the forest plot under dominant comparison (CC + CG vs. GG).

CG vs. GG Study	Experimental Events	Experimental Total	Control Events	Control Total	OR	95%-CI	Weight (fixed)	Weight (random)
**Ethnicity = Caucasian**								
Kuschel et al. 2002 (a)	457	912	318	657	1.07	[0.88; 1.31]	3.6%	2.4%
Kuschel et al. 2002 (b)	303	581	318	657	1.16	[0.93; 1.45]	2.8%	2.2%
Sanyal et al. 2004	140	254	134	250	1.06	[0.75; 1.51]	1.2%	1.6%
Forsti et al. 2004	110	197	152	272	1.00	[0.69; 1.44]	1.1%	1.5%
Broberg et al. 2005	36	57	67	130	1.61	[0.85; 3.05]	0.3%	0.7%
Festa et al. 2005	111	207	266	496	1.00	[0.72; 1.38]	1.4%	1.7%
Auranen et al. 2005 (a)	335	642	372	741	1.08	[0.88; 1.34]	3.3%	2.3%
Millikan et al. 2005 (a)	566	1154	503	1021	0.99	[0.84; 1.17]	5.4%	2.6%
Auranen et al. 2005 (b)	134	275	169	331	0.91	[0.66; 1.25]	1.6%	1.7%
Auranen et al. 2005 (c)	130	272	359	742	0.98	[0.74; 1.29]	2.0%	1.9%
Auranen et al. 2005 (d)	109	233	318	657	0.94	[0.69; 1.26]	1.7%	1.8%
Wu et al. 2006	283	537	259	541	1.21	[0.96; 1.54]	2.4%	2.2%
Lu et al. 2006	198	369	281	488	0.85	[0.65; 1.12]	2.2%	2.0%
Thirumaran et al. 2006	229	455	221	471	1.15	[0.89; 1.48]	2.1%	2.0%
Zienolddiny et al. 2006	155	281	165	327	1.21	[0.88; 1.66]	1.3%	1.7%
Ryk et al. 2006	105	177	80	152	1.31	[0.85; 2.03]	0.7%	1.2%
Tarasov et al. 2006	67	135	88	166	0.87	[0.55; 1.38]	0.8%	1.2%
Figueroa et al. 2007	449	965	407	918	1.09	[0.91; 1.31]	4.4%	2.5%
Pardini et al. 2008	236	482	220	459	1.04	[0.81; 1.35]	2.3%	2.1%
Mosor et al. 2008	63	129	134	245	0.79	[0.52; 1.21]	0.9%	1.3%
Ziolkowska et al. 2008	69	158	93	175	0.68	[0.44; 1.05]	1.0%	1.3%
Smith et al. 2008 (a)	127	290	176	358	0.81	[0.59; 1.10]	1.7%	1.8%
Margulis et al. 2009	142	279	160	312	0.98	[0.71; 1.36]	1.5%	1.7%
Bastos et al. 2009	44	100	82	194	1.07	[0.66; 1.75]	0.6%	1.1%
Desjardins et al. 2009	34	88	33	67	0.65	[0.34; 1.23]	0.5%	0.7%
Schuetz et al. 2009 (a)	72	148	270	525	0.89	[0.62; 1.29]	1.2%	1.5%
Schuetz et al. 2009 (b)	62	146	270	525	0.70	[0.48; 1.01]	1.3%	1.5%
Schuetz et al. 2009 (c)	33	61	270	525	1.11	[0.65; 1.89]	0.5%	1.0%
Rajaraman et al. 2010	209	465	213	424	0.81	[0.62; 1.05]	2.4%	2.0%
Silva et al. 2010	112	137	219	271	1.06	[0.63; 1.80]	0.5%	1.0%
Jelonek et al. 2010 (a)	62	117	71	131	0.95	[0.58; 1.57]	0.6%	1.0%
Jelonek et al. 2010 (b)	47	86	54	98	0.98	[0.55; 1.76]	0.5%	0.8%
Jelonek et al. 2010 (c)	43	83	208	368	0.83	[0.51; 1.33]	0.7%	1.1%
Betti et al. 2011	55	120	100	231	1.11	[0.71; 1.73]	0.7%	1.2%
Erculj et al. 2012	10	13	13	17	1.03	[0.19; 5.66]	0.1%	0.1%
Gil et al. 2012	66	118	43	87	1.30	[0.75; 2.26]	0.4%	0.9%
Mosor et al. 2013	92	188	134	245	0.79	[0.54; 1.16]	1.2%	1.4%
Smolkova et al. 2014	203	414	241	493	1.01	[0.77; 1.31]	2.2%	2.0%
Goricar et al. 2015	68	79	340	373	0.60	[0.29; 1.25]	0.3%	0.6%
Uzunoglu et al. 2016	39	82	44	99	1.13	[0.63; 2.04]	0.4%	0.8%
Psoma et al. 2019	5	14	20	48	0.78	[0.23; 2.67]	0.1%	0.2%
Dimitrakopoulos et al. 2021	78	126	67	128	1.48	[0.90; 2.44]	0.5%	1.0%
Minina et al. 2022	96	123	121	142	0.62	[0.33; 1.16]	0.5%	0.7%
**Fixed effect model**		**11749**		**15557**	**1.00**	**[0.95; 1.05]**	**61.1%**	--
**Random effect model**					**1.00**	**[0.95; 1.05]**	--	**62.2%**
Heterogeneity: $I^2 = 0\%$, $\tau^2 = 0$, $p = 0.6009$								
Test for effect in subgroup (random effects): $z = 0.11$ ($p = 0.9136$)								
**Ethnicity = Mixed**								
Millikan et al. 2005 (b)	287	727	240	640	1.09	[0.87; 1.35]	3.0%	2.3%
Shen et al. 2006	199	427	260	505	0.82	[0.64; 1.06]	2.5%	2.0%
Hebbring et al. 2006 (a)	94	176	79	168	1.29	[0.85; 1.97]	0.7%	1.3%
Hebbring et al. 2006 (b)	63	103	79	168	1.77	[1.08; 2.92]	0.5%	1.0%
Choudhury et al. 2008	332	679	330	705	1.09	[0.88; 1.34]	3.3%	2.3%
Smith et al. 2008 (b)	24	49	22	68	2.01	[0.94; 4.28]	0.2%	0.6%
Jiao et al. 2012 (a)	89	150	71	141	1.44	[0.90; 2.29]	0.6%	1.1%
Jiao et al. 2012 (b)	160	327	247	422	0.68	[0.51; 0.91]	2.2%	1.9%
Wu et al. 2013	130	162	152	190	1.02	[0.60; 1.72]	0.5%	1.0%
**Fixed effect model**		**2800**		**3007**	**1.03**	**[0.93; 1.15]**	**13.5%**	--
**Random effect model**					**1.10**	**[0.90; 1.35]**	--	**13.5%**
Heterogeneity: $I^2 = 64\%$, $\tau^2 = 0.0593$, $p = 0.0050$								
Test for effect in subgroup (random effects): $z = 0.91$ ($p = 0.3654$)								
**Ethnicity = Asian**								
Zhang et al. 2005	110	196	149	266	1.00	[0.69; 1.46]	1.1%	1.5%
Lan et al. 2005	61	81	54	64	0.56	[0.24; 1.31]	0.3%	0.5%
Maria et al. 2009	497	1008	502	1045	1.05	[0.88; 1.25]	4.9%	2.5%
Fan et al. 2010	105	221	214	449	0.99	[0.72; 1.37]	1.5%	1.7%
Zheng et al. 2011	323	469	361	644	1.73	[1.35; 2.23]	1.9%	2.1%
Zheng et al. 2011	158	242	201	357	1.46	[1.04; 2.05]	1.1%	1.6%
Jiang et al. 2011	83	109	181	300	2.10	[1.28; 3.45]	0.5%	1.0%
Huang et al. 2012	427	671	457	612	0.59	[0.47; 0.76]	3.4%	2.1%
Li et al. 2013	210	273	298	490	2.15	[1.54; 3.00]	1.0%	1.6%
Jin et al. 2015	96	148	171	298	1.37	[0.91; 2.06]	0.8%	1.3%
Zhang et al. 2015	233	413	248	515	1.39	[1.07; 1.81]	1.9%	2.0%
Li et al. 2015	399	617	451	729	1.13	[0.90; 1.41]	2.9%	2.2%
Hu et al. 2018	185	290	183	294	1.07	[0.76; 1.50]	1.3%	1.6%
Chen et al. 2020	399	617	451	729	1.13	[0.90; 1.41]	2.9%	2.2%
Zehtab et al. 2022	26	26	22	22	1.18	[0.02; 61.78]	0.0%	0.0%
**Fixed effect model**		**5381**		**6814**	**1.16**	**[1.08; 1.25]**	**25.4%**	--
**Random effect model**					**1.20**	**[1.00; 1.45]**	--	**24.3%**
Heterogeneity: $I^2 = 80\%$, $\tau^2 = 0.0971$, $p < 0.0001$								
Test for effect in subgroup (random effects): $z = 1.92$ ($p = 0.0550$)								
**Fixed effect model**		**19930**		**25378**	**1.05**	**[1.01; 1.09]**	**100.0%**	--
**Random effect model**					**1.05**	**[0.98; 1.11]**	--	**100.0%**

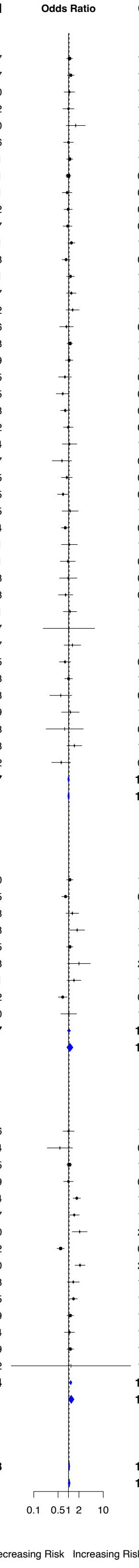

Heterogeneity: $I^2 = 53\%$, $\tau^2 = 0.0322$, $p < 0.0001$

Test for overall effect (fixed effect): $z = 2.34$ ($p = 0.0191$)

Test for overall effect (random effects): $z = 1.39$ ($p = 0.1631$)

Test for subgroup differences (fixed effect): $\chi^2_2 = 10.38$, df = 2 ($p = 0.0056$)

Test for subgroup differences (random effects): $\chi^2_2 = 3.89$, df = 2 ($p = 0.1433$)

Figure S4. The association between rs1805794 polymorphism and cancer risk by showing the forest plot under heterozygote comparison (CG vs. GG).

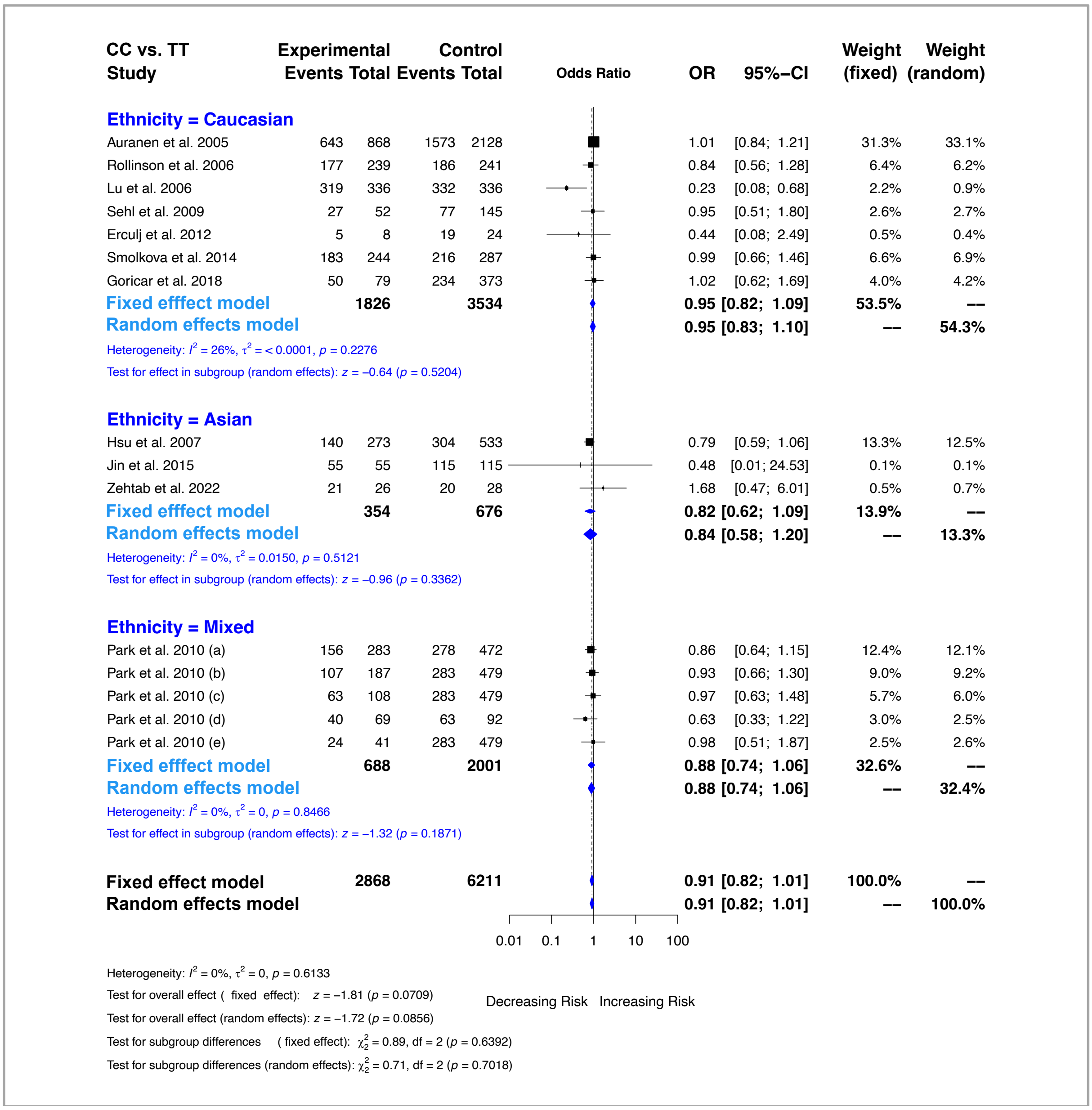

Figure S5. The association between rs709816 polymorphism and cancer risk by showing the forest plot under homozygote comparison (CC vs. TT).

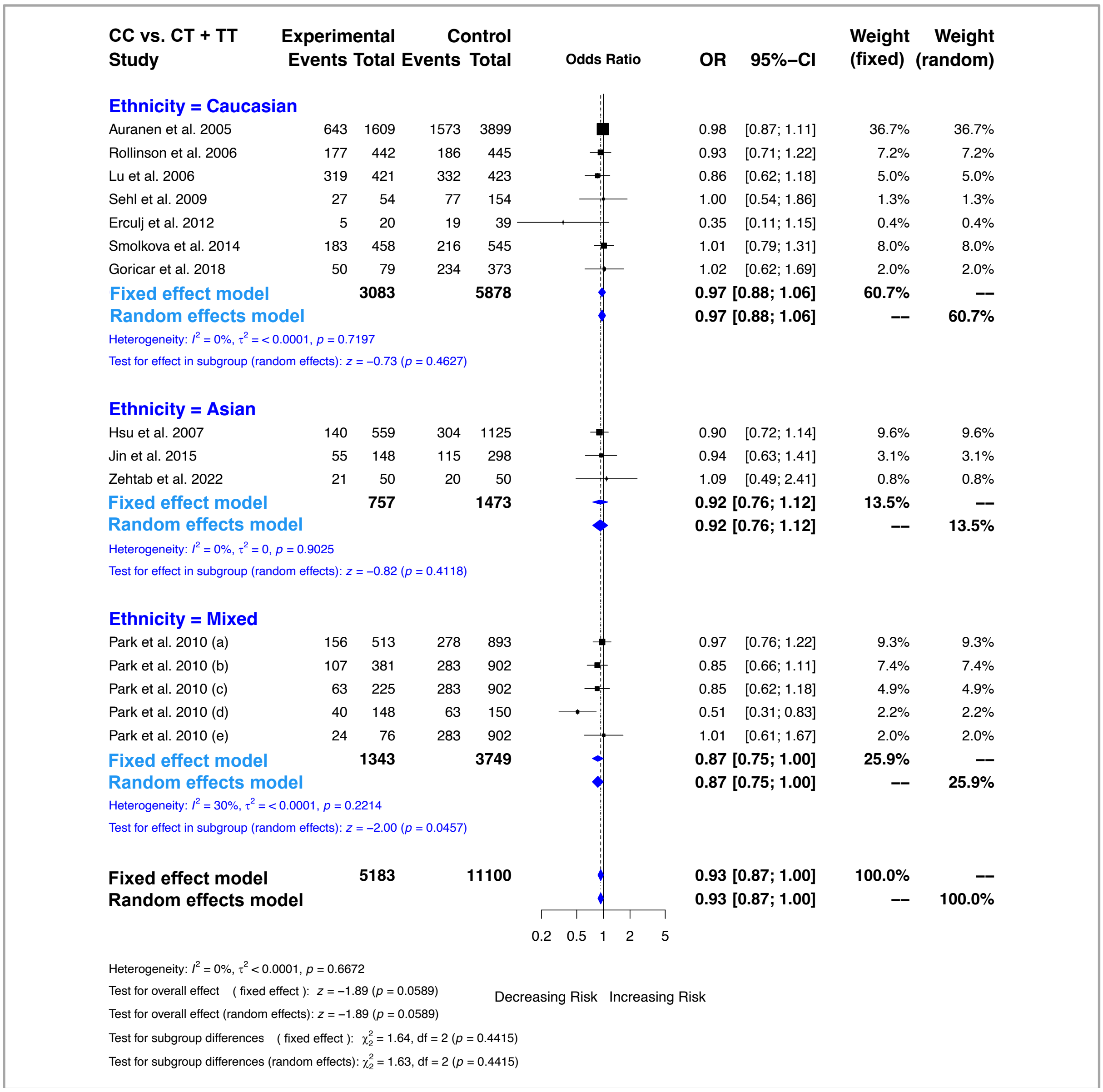

Figure S6. The association between rs709816 polymorphism and cancer risk by showing the forest plot under recessive comparison (CC vs. CT + TT).

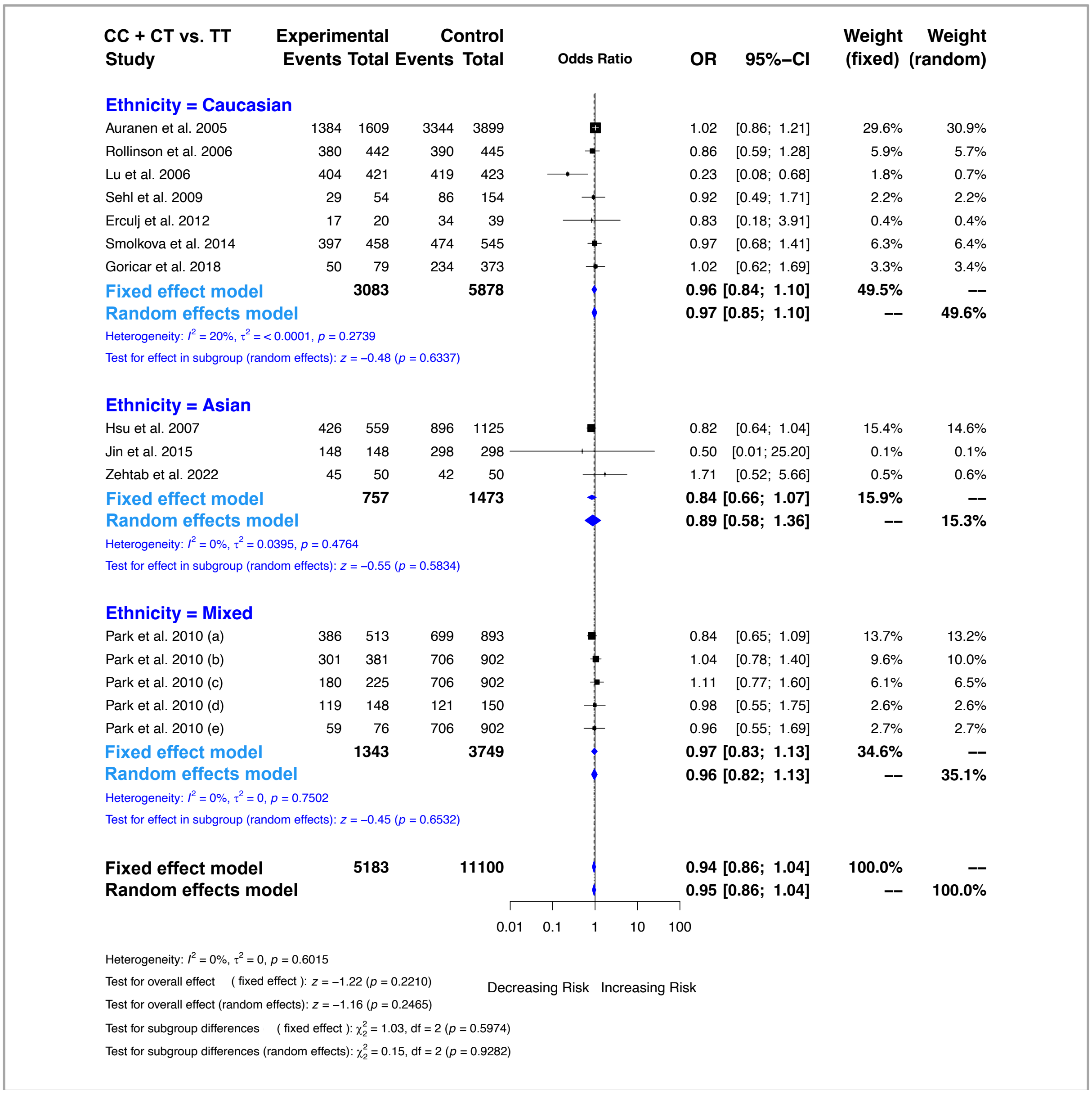

Figure S7. The association between rs709816 polymorphism and cancer risk by showing the forest plot under dominant comparison (CC + CT vs. TT).

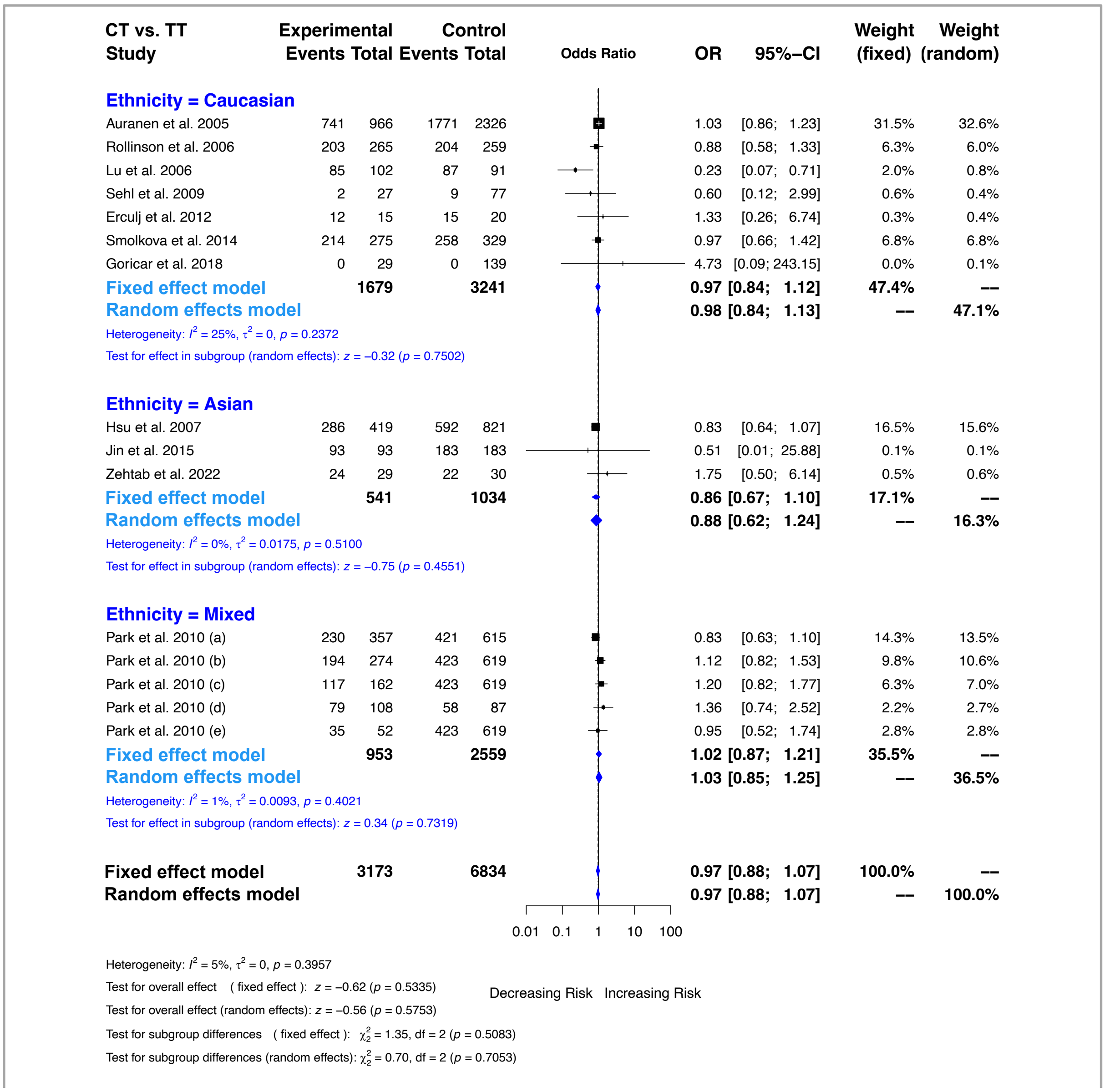

Figure S8. The association between rs709816 polymorphism and cancer risk by showing the forest plot under heterozygote comparison (CT vs. TT).

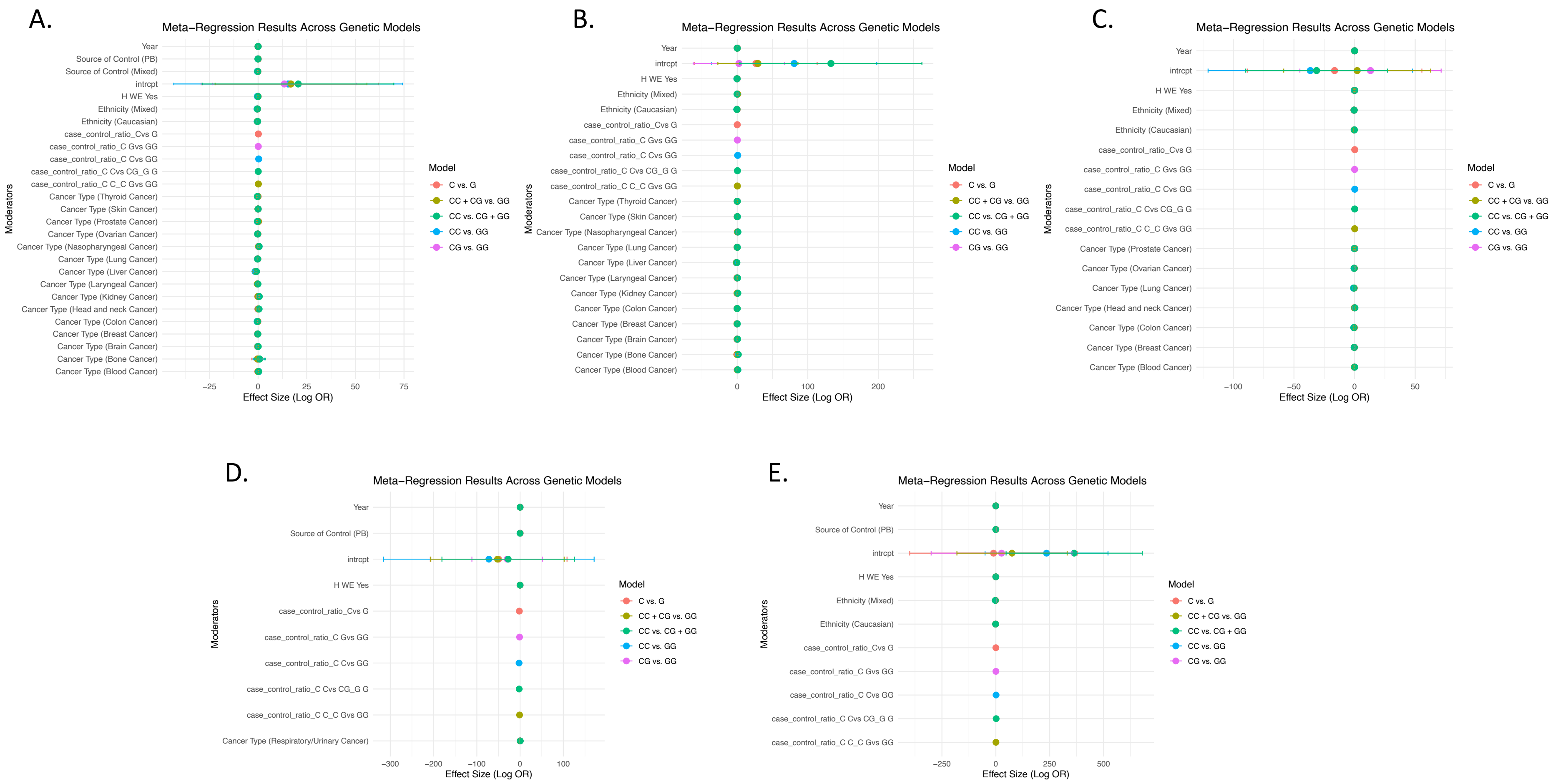

Figure S9. Meta-regression analysis results show the estimated effects of key moderators on effect size. Each point represents a moderator, with 95% confidence intervals (CIs) shown as error bars. The reference category for each categorical variable is omitted and used as the baseline. A confidence interval crossing zero indicates a non-significant effect. The analysis was conducted for five genetic models (C vs. G, CC vs. GG, CG vs. GG, CC + CG vs. GG, CC vs. CG + GG) to identify potential sources of heterogeneity for the rs1805794 polymorphism with subgroups of (A) overall cancer risks, (B) HB, (C) PB sources of control populations, (D) Asian populations, and (E) blood cancer.

References

[1] X. Li, W.D. Heyer, Homologous recombination in DNA repair and DNA damage tolerance, Cell Res. 18 (2008). https://doi.org/10.1038/cr.2008.1.

[2] Y.Z. Chen, D. Zuo, H.L. Ren, S.J. Fan, G. Ying, Bioinformatics Analysis of Expression and Alterations of BARD1 in Breast Cancer, Technol. Cancer Res. Treat. 18 (2019). https://doi.org/10.1177/1533033819892260.

[3] A. Rangel-Pozzo, S. Liu, G. Wajnberg, X. Wang, R.J. Ouellette, G.G. Hicks, D. Drachenberg, S. Mai, Genomic Analysis of Localized High-Risk Prostate Cancer Circulating Tumor Cells at the Single-Cell Level, Cells. 9 (2020). https://doi.org/10.3390/cells9081863.

[4] A. Baudot, V. De La Torre, A. Valencia, Mutated genes, pathways and processes in tumours, EMBO Rep. 11 (2010). https://doi.org/10.1038/embor.2010.133.

[5] M.H. Schaefer, L. Serrano, Cell type-specific properties and environment shape tissue specificity of cancer genes, Sci. Rep. 6 (2016). https://doi.org/10.1038/srep20707.

[6] A. Cabrera-Andrade, A. López-Cortés, G. Jaramillo-Koupermann, C. Paz-Y-miño, Y. Pérez-Castillo, C.R. Munteanu, H. González-Díaz, A. Pazos, E. Tejera, Gene prioritization through consensus strategy, enrichment methodologies analysis, and networking for osteosarcoma pathogenesis, Int. J. Mol. Sci. 21 (2020). https://doi.org/10.3390/ijms21031053.

[7] H. Lee, S.W. Kong, P.J. Park, Integrative analysis reveals the direct and indirect interactions between DNA copy number aberrations and gene expression changes, Bioinformatics. 24 (2008). https://doi.org/10.1093/bioinformatics/btn034.

[8] Y.J. Xia, J. Zhao, C. Yang, Identification of key genes and pathways for melanoma in the TRIM family, Cancer Med. 9 (2020). https://doi.org/10.1002/cam4.3545.

[9] C. SHEN, Y. WANG, Z. WU, L. DA, S. GAO, L. XIE, Y. QIE, Y. WANG, Z. ZHANG, D. TIAN, H. HU, Long noncoding RNAs, ENST00000598996 and ENST00000524265, are correlated with favorable prognosis and act as potential tumor suppressors in bladder cancer, Oncol. Rep. 44 (2020). https://doi.org/10.3892/or.2020.7733.

[10] L. Bian, Y. Meng, M. Zhang, D. Li, MRE11-RAD50-NBS1 complex alterations and DNA damage response: Implications for cancer treatment, Mol. Cancer. 18 (2019) 1–14. https://doi.org/10.1186/s12943-019-1100-5.

[11] L. Zhang, H. Wu, X. Xiao, K. Li, Y. Zhang, L. Zhang, T. Wen, Analysis on regulatory network linked to Hpa gene in invasion and metastasis of colon cancer, Saudi J. Biol. Sci. 24 (2017). https://doi.org/10.1016/j.sjbs.2017.01.019.

[12] J. He, J. Yang, W. Chen, H. Wu, Z. Yuan, K. Wang, G. Li, J. Sun, L. Yu, Molecular features of triple negative breast cancer: Microarray evidence and further integrated analysis, PLoS One. 10 (2015). https://doi.org/10.1371/journal.pone.0129842.

[13] S.J. Furney, D.G. Higgins, C.A. Ouzounis, N. López-Bigas, Structural and functional properties of genes involved in human cancer, BMC Genomics. 7 (2006). https://doi.org/10.1186/1471-2164-7-3.

[14] Y. Zhang, F. Yang, Analyzing the disease module associated with osteosarcoma via a network- and pathway-based approach, Exp. Ther. Med. 16 (2018). https://doi.org/10.3892/etm.2018.6506.

[15] A. Cerbinskaite, A. Mukhopadhyay, E.R. Plummer, N.J. Curtin, R.J. Edmondson, Defective homologous recombination in human cancers, Cancer Treat. Rev. 38 (2012) 89–100. https://doi.org/10.1016/j.ctrv.2011.04.015.

[16] M. Shao, W. Li, S. Wang, Z. Liu, Identification of key genes and pathways associated with esophageal squamous cell carcinoma development based on weighted gene correlation network analysis, J. Cancer. 11 (2020). https://doi.org/10.7150/jca.30699.
